# Inhibition of the RacGEF VAV3 by the small molecule IODVA1 impedes RAC signaling and overcomes resistance to tyrosine kinase inhibition in acute lymphoblastic leukemia

**DOI:** 10.1038/s41375-021-01455-3

**Published:** 2021-10-28

**Authors:** Shailaja Hegde, Anjelika Gasilina, Mark Wunderlich, Yuan Lin, Marcel Buchholzer, Oliver H. F. Krumbach, Mohammad Akbarzadeh, Mohammad Reza Ahmadian, William Seibel, Yi Zheng, John P. Perentesis, Benjamin E. Mizukawa, Lisa Privette Vinnedge, José A. Cancelas, Nicolas N. Nassar

**Affiliations:** 1 Division of Experimental Hematology and Cancer Biology, Children’s Hospital Research Foundation, 3333 Burnet Ave, Cincinnati, OH 45229 USA; 2 Hoxworth Blood Center, University of Cincinnati Academic Health Center, Cincinnati, OH USA; 3 Institute of Biochemistry and Molecular Biology II, Medical Faculty, Heinrich-Heine-University, Düsseldorf, 40225 Germany; 4 Division of Oncology, Cincinnati Children’s Hospital Medical Center, Cancer and Blood Diseases Institute, 3333 Burnet Ave, Cincinnati, OH 45229 USA; 5 Department of Pediatrics, University of Cincinnati College of Medicine, 3230 Eden Ave, Cincinnati, OH 45267 USA

**Keywords:** Acute lymphocytic leukaemia, Biochemistry

## Abstract

Aberrant RHO guanine nucleotide exchange factor (RhoGEF) activation is chief mechanism driving abnormal activation of their GTPase targets in transformation and tumorigenesis. Consequently, a small-molecule inhibitor of RhoGEF can make an anti-cancer drug. We used cellular, mouse, and humanized models of RAC-dependent BCR-ABL1-driven and Ph-like acute lymphoblastic leukemia to identify VAV3, a tyrosine phosphorylation–dependent RacGEF, as the target of the small molecule IODVA1. We show that through binding to VAV3, IODVA1 inhibits RAC activation and signaling and increases pro-apoptotic activity in BCR-ABL1-transformed cells. Consistent with this mechanism of action, cellular and animal models of BCR-ABL1-induced leukemia in *Vav3*-null background do not respond to IODVA1. By durably decreasing in vivo RAC signaling, IODVA1 eradicates leukemic propagating activity of TKI-resistant BCR-ABL1(T315I) B-ALL cells after treatment withdrawal. Importantly, IODVA1 suppresses the leukemic burden in the treatment refractory pediatric Ph^+^ and TKI-resistant Ph^+^ B-ALL patient-derived xenograft models better than standard-of-care dasatinib or ponatinib and provides a more durable response after treatment withdrawal. Pediatric leukemia samples with diverse genetic lesions show high sensitivity to IODVA1 ex vivo and this sensitivity is VAV3 dependent. IODVA1 thus spearheads a novel class of drugs that inhibits a RacGEF and holds promise as an anti-tumor therapy.

## Introduction

RAC GTPases have been associated with pro-tumorigenic functions and development of cancer. They are pivotal in most aggressive types of leukemias [1–6] and are characteristic of resistance to chemo-, radio-, and targeted-therapies and associated with persistence of leukemic stem cells [7–10]. Reducing RAC activity specifically in cancer cells is desirable; however, no small-molecule inhibitor of RAC signaling is in clinical use despite many efforts. Therefore, finding a small-molecule inhibitor of RAC or of its upstream activator will provide an effective strategy for treatment of malignancies, especially leukemia.

We recently identified the small molecule IODVA1, a 2-guanidinobenzimidazole derivative with anti-tumorigenic properties. Initial characterization of IODVA1 ruled out kinase inhibition potential but showed inhibition of RAC activity and signaling at low concentrations and within minutes of exposure [11]. Here, we use in vitro and in vivo leukemic models of the chimeric BCR-ABL1 oncoprotein B-cell acute lymphoblastic leukemia (Ph^+^ B-ALL) and Ph-like to reveal the mechanism of action of IODVA1. Expression of p190- or p210-BCR-ABL1 or Ph-like rearrangements activates RAC signaling pathways to regulate leukemogenesis [5] and deleting RAC isoforms alone impairs leukemogenesis induced by p190- or p210-BCR-ABL1 expression in the hematopoietic stem and progenitor cell compartment [1, 2, 12, 13]. Genetic deletion of the RAC guanine nucleotide exchange factor (RacGEF) VAV3 inhibits leukemic cell survival in vitro and in vivo and sensitizes lymphoblastic leukemia cells to tyrosine kinase inhibitors (TKIs) [12]. In addition, despite tremendous success of TKIs in treating B-ALL in the clinic, the response is incomplete [14–17]. Thus, there is an unmet need for novel treatment options for patients with TKI-resistant leukemia and treatments that prevent leukemic-cell persistence.

Here, we show that by binding to VAV3, IODVA1 inhibits RAC activation and its downstream signaling and induces apoptosis specifically in leukemic cells in vivo and in vitro. IODVA1 monotherapy is superior to ABL1-TKIs dasatinib or ponatinib in murine and pediatric patient-derived xenograft models of wild-type and TKI-resistant Ph^+^ BCR-ABL1. We also show that IODVA1 exerts anti-proliferative effects on samples derived from patients with relapsed and de novo ALL leukemias in a VAV3-dependent manner. Our findings have direct implications for overcoming TKI resistance in the clinic and for treating cancers where VAV3 is a target, including RAS-driven cancers, making IODVA1 a promising first-in-class drug candidate.

## Results

### IODVA1 specifically targets BCR-ABL1 B-ALL cells in vitro

**Fig. 1:**
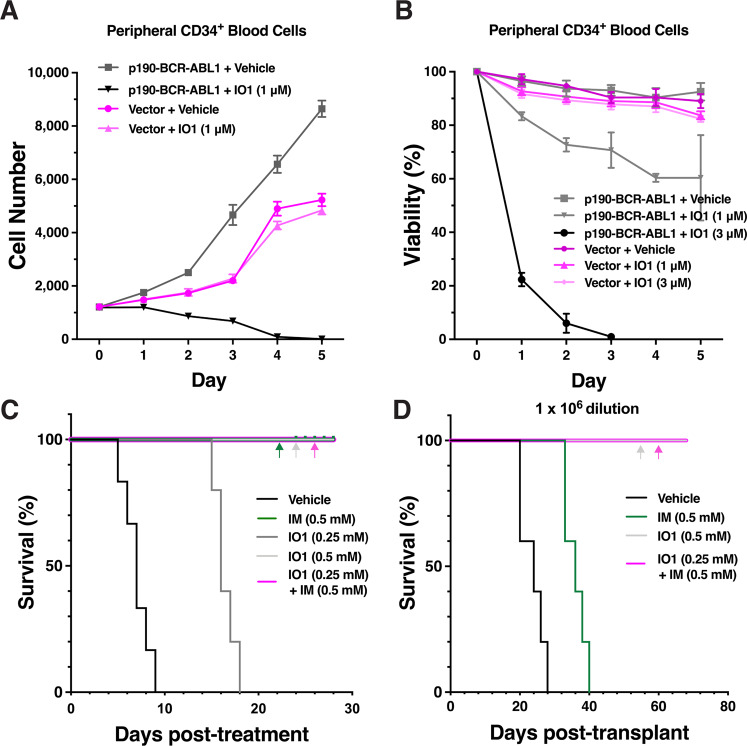
IODVA1 inhibits the proliferation and survival of BCR-ABL1-driven cells in vitro and in vivo and eradicates leukemia-propagating cells in secondary transplants. **A** Cell proliferation of human peripheral CD34^+^ blood cells transduced with p190-BCR-ABL1 (gray line, squares, and black line, downward triangles) or empty vector (lilac lines, circles, and upward triangles) virus and treated with either vehicle or IODVA1 (IO1, 1 μM). Live cells were counted by flow cytometry (GFP^+^/7-AAD^-^). Mean ± SD of a representative experiment done in triplicates. **B** Cells were transduced and cultured as in (**A**) but incubated with either vehicle or IODVA1 (IO1, 1 or 3 μM) and viability (%) was determined by trypan blue exclusion. Mean ± SD of a representative experiment done in triplicates. **C** Kaplan–Meier survival plot of p190-BCR-ABL1 leukemic mice post treatment with vehicle control, IODVA1 (IO1), imatinib (IM), or the combination at the indicated concentrations in the pump. LDBM cells were transduced with p190-BCR-ABL1/EGFP retrovirus and transplanted into recipient mice. After initial assessment of leukemic burden, drugs were delivered for 28 days in subcutaneously implanted osmotic pumps at day 23 post leukemia transplantation. *N* = 5 mice per group, except for vehicle *N* = 6. **D** Kaplan–Meier survival plot of secondary mice transplants with the 10^6^-cell dilution. Bone marrow cells from mice treated with vehicle, IM, IODVA1 (IO1), or the combination at the indicated concentrations were transplanted into secondary recipients. *N* = 5 mice per group. Colored arrows indicate overlapping corresponding curves.

First, we assessed whether IODVA1 is specific to oncogene-transformed cells. Treatment with IODVA1 (IO1, 1 μM) reduced the number of live CD34^+^ cells expressing p190-BCR-ABL1, but not empty vector (Fig. 1A). Viability of p190-BCR-ABL1-, but not vector-transduced CD34^+^ cells decreased in a dose-dependent manner (Fig. 1B). IODVA1 irreversibly inhibited the survival of p190- and p210-BCR-ABL1 but not of empty vector-expressing Ba/F3 cells with EC_50_ of 380 nM, NALM-1 cells with an EC_50_ of 680 nM and inhibited the colony-forming ability of BCR-ABL1-Ba/F3 cells (Supplementary Fig. S1A–D). Together, these results indicate that IODVA1 specifically targets proliferation and survival of BCR-ABL1-transformed cells and are consistent with our previous report that IODVA1 targets transformed cells [11].

### IODVA1 decreases in vivo leukemic burden and prevents leukemia-related death

We next assessed in vivo efficacy of IODVA1 on a murine model of p190-BCR-ABL1-induced B-ALL and compared it to ABL1-TKI imatinib. C57Bl/10 mice were transplanted with p190-BCR-ABL1 low-density bone marrow (LDBM) cells, stratified into five groups post leukemia development, and administered either vehicle, 0.25 or 0.5 mM IODVA1, 0.5 mM imatinib, or the combination 0.25 mM IODVA1 + 0.5 mM imatinib via osmotic pumps. The low IODVA1 dose increased survival by an average of 10 days compared to vehicle-treated mice (Fig. 1C). Administration of imatinib, the higher IODVA1 dose, or the combination prevented leukemia-related death during treatment. Importantly, IODVA1 decreased the residual leukemic progenitor B cells (EGFP^+^/B220^+^) from peripheral blood (PB) of treated mice (Supplementary Fig. S1E).

### IODVA1 eradicates leukemic propagating activity

Despite its significant clinical success, imatinib and, more generally, TKIs do not eliminate leukemic stem/progenitor cells in the bone marrow (BM), which can lead to residual disease, resistance, and relapse [18, 19]. To determine if IODVA1 eradicates progenitor tumor-propagating capacity, as a functional surrogate of minimal residual disease capable of leukemia relapse, leukemic animals treated with vehicle, imatinib, 0.5 mM IODVA1, or the combination (Fig. 1C) were sacrificed and their BM cells were transplanted into secondary recipients in a limiting dilution series of 1 × 10^6^, 0.3 × 10^6^, and 0.1 × 10^6^ cell doses and monitored for leukemia development and survival in the absence of any additional therapy. Data for the 10^6^-cell dilution transplant indicate that administration of IODVA1 alone or in combination with imatinib resulted in survival of recipients beyond the 70-day endpoint analysis (Fig. 1D). Mice transplanted with BM cells from primary recipient mice treated with imatinib alone died by day 40 post transplantation. Analysis at week 5 post transplantation of the leukemic progenitor cells (EGFP^+^/B220^+^) from the PB of secondary transplant mice indicates that IODVA1 is better than imatinib at eradicating leukemic cell burden (Supplementary Fig. S1F). Poisson’s distribution analysis of the lower cell dose transplantations (Supplementary Fig. S1G–J) indicates >10-fold depletion of tumor-propagating activity in grafts from IODVA1– or IODVA1 + imatinib-treated leukemic mice compared with leukemic mice treated with imatinib alone.

### IODVA1 eradicates TKI-resistant BCR-ABL1 B-ALL

**Fig. 2:**
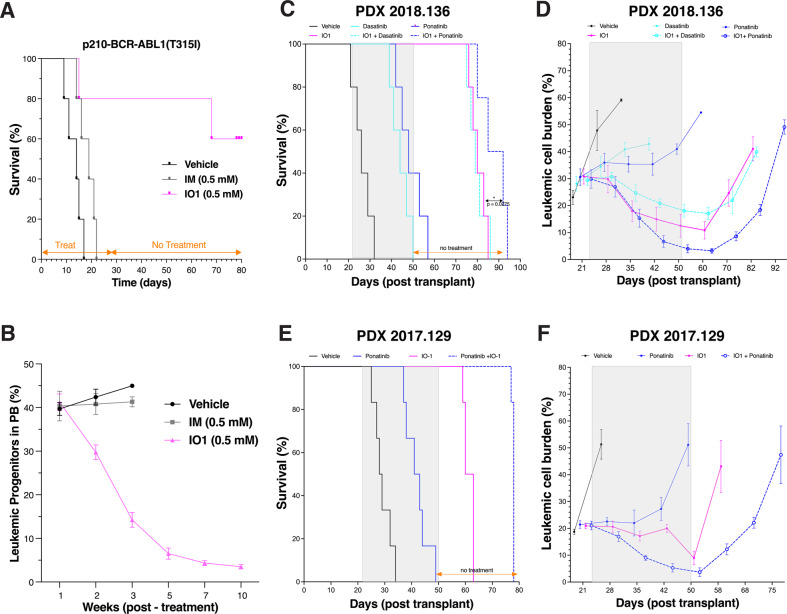
IODVA1 increases the survival of PDX models of Ph^+^ B-ALL better than ABL1-TKIs. Mice engrafted with LDBM cells transduced with TKI-resistant p210-BCR-ABL1(T315I) gatekeeper mutant. Treatment was delivered continuously for 28 days via surgically implanted subcutaneous pumps (*N* = 5 per treatment group). After 28 days, mice were monitored without any additional treatment. **A** Kaplan–Meier survival plot of mice with imatinib-resistant leukemia. Pumps either carried vehicle control (black line), 0.5 mM imatinib (IM, gray line), or 0.5 mM IODVA1 (IO1, lilac line). **B** Percentage of leukemic progenitors (EGFP^+^) B cells in peripheral blood (PB) was assessed by flow cytometry at the indicated week and plotted. Only IODVA1-treated mice remained alive for analysis at weeks 5, 7, and 10. **C**, **D** NSG mice engrafted with B-ALL patient sample 2018-136 were treated days 22–50 with vehicle (black line), IODVA1 (solid lilac line), dasatinib (solid cyan line), IODVA1 + dasatinib (dashed cyan line), ponatinib (solid blue line), IODVA1 + ponatinib (dashed blue line). Treatment duration is highlighted by a gray rectangle. *N* = 5 mice per treatment group. **C** Kaplan–Meier survival plot of PDX 2018-136-engrafted mice during treatment and after treatment withdrawal. **D** Leukemic cell burden assessment in the PB of PDX-implanted mice at the indicated time points. **E**, **F** Patient sample 2017-129 was engrafted as in (**C**) and (**D**), except mice were treated only with vehicle, IODVA1, ponatinib and IODVA1 + ponatinib combination.

We have reported that IODVA1 has no inhibitory activity in vitro against major wild-type kinases including ABL1 and SRC-like kinases [11]. We confirmed that it does not target BCR-ABL1 by immuno-precipitating it from p190-BCR-ABL1-Ba/F3 cells treated with vehicle control, imatinib, or IODVA1 (3 μM) and probing its phosphotyrosine levels by immunoblotting. We also assessed levels of phospho-Crkl, a marker for BCR-ABL1 transformation and activity. Unlike imatinib, IODVA1 treatment did not affect the phosphorylation level of p190-BCR-ABL1 and of pCrkl (Supplementary Fig. S2). Thus, the anti-proliferative activity of IODVA1 toward in vitro and in vivo BCR-ABL1 B-ALL models and its ability to eradicate residual disease cannot be explained by ABL1 inhibition. To further test this idea, we evaluated efficacy of IODVA1 in p210-BCR-ABL1(T315I) gatekeeper mutant leukemia model—one of the most common compound mutations arising in patients on imatinib therapy [20]. While imatinib-treated mice died by day 22, before the end of the treatment (Fig. 2A, IM), 80% of IODVA1-treated mice survived until day 65, or 37 days post treatment withdrawal. In all, 60% of IODVA1-treated mice survived until the end of the experiment at day 80, or 52 days post treatment withdrawal (Fig. 2A, IO1). IODVA1 treatment alone significantly decreased leukemic progenitor levels by 24% by week 2, by 84% by week 5, and by 91% by week 10 (Fig. 2B).

### IODVA1 decreases survival of patient-derived leukemia cells in vitro and in vivo

Cells from PDX models representing pediatric Ph^+^, Ph-like, and MLL-rearranged B-ALL patients including patients with TKI-resistant BCR-ABL1(T315I) mutation (Supplementary Table S1) were found in general to be highly sensitive to IODVA1 ex vivo (Supplementary Fig. S3A–C). To validate these survival results in vivo, we transplanted patient 2018-136 cells with relapsed Ph^+^ (BCR-ABL1), IKZF1, ΔCDKN2A/B, and ΔPAX5 leukemia into NSG mice, stratified mice into six groups (five mice per group) post leukemic development and administered either vehicle, IODVA1 (4 mM), dasatinib or ponatinib (0.5 mM), and the IODVA1 + dasatinib or IODVA1 + ponatinib combinations via subcutaneous osmotic pumps for 4 weeks. Data show that all control group mice died at day 32 post transplantation, or 10 days post administration of the vehicle control (Fig. 2C). The dasatinib- and ponatinib-treated mice died by day 50 and 57, or 28 and 35 days after treatment began, respectively. Mice treated with IODVA1 survived until day 85, or 35 days post treatment withdrawal. There was no difference in survival between IODVA1 or IODVA1 + dasatinib-treated mice. The IODVA1 + ponatinib-treated mice survived until day 94, or 44 days post treatment withdrawal, showing that this combination is superior to IODVA1 alone (*p* = 0.0275). Unlike ponatinib or dasatinib monotherapies, PB leukemic progenitor cell counts dropped from 30.8 ± 2.8% at the beginning (day 22) to 12.5 ± 4.2% at the end of the treatment in IODVA1 monotherapy (day 50, Fig. 2D). This level was unchanged at day 60 but steadily increased after that to reach 41 ± 4.5% at day 82. The IODVA1 + ponatinib combination therapy was the most efficient at decreasing the leukemic progenitor levels to 4.0 ± 1.5% at the end of the treatment (day 50). This level kept decreasing to 3.3 ± 1% 10 days post treatment arrest (day 60) but steadily increased albeit at a lower rate to reach 49.1 ± 2.6% at day 92.

To confirm that IODVA1 also overcomes TKI resistance in a PDX-derived system, we used NSG mice engrafted with sample from patient 2017-129 with Ph^+^ (BCR-ABL1; T315I) and mutated *SETD2*, *SF3B1*, and *TP53* with disease relapse after initial treatment. In vitro, these cells showed sensitivity to IODVA1 and no sensitivity to dasatinib, the JAK inhibitor ruxolitinib, or dasatinib/ruxolitinib combination (Supplementary Fig. S3A). Mice were stratified into four groups and administered either vehicle, IODVA1 (4 mM), ponatinib (0.5 mM), and the IODVA1 + ponatinib combination via osmotic pumps for 4 weeks after which treatment was stopped (Fig. 2E). Vehicle-treated mice died between day 25 and 34. Ponatinib-treated mice survived significantly longer but died before treatment ended between day 37 and 49. IODVA1-treated mice died between day 59 and 63, or 9 and 13 days after treatment ended. Interestingly, the IODVA1/ponatinib combination therapy increased animal survival the most as death occurred between day 77 and 78, or 27 and 28 days after treatment ended. Leukemic cell burden analysis (Fig. 2F) shows that ponatinib does not decrease levels of leukemic cells in the PB. IODVA1 on the other hand decreases the leukemic burden by 57%, from 21.0 ± 1.0% at the beginning of the treatment (day 21) to 9.0 ± 2.5% at the end of the treatment (day 50). However, the IODVA1-treated mice still die from leukemia as the leukemic burden increases to 43.1 ± 9.7% 8 days after treatment ended. The combo treatment decreased the leukemic burden 5.7-fold, from 21 ± 1.6% to 3.7 ± 1.6%, at the end of the treatment (day 50). However, the residual leukemic burden increased with time eventually leading to death.

Taken together, our in vivo PDX data show that IODVA1 is superior to standard-of-care drugs dasatinib and ponatinib and that IODVA1 and ponatinib synergize to prolong survival even after treatment has stopped, although they do not completely eradicate therapy-refractory multi-lesion leukemia.

### IODVA1 decreases RAC activity and downstream signaling

**Fig. 3:**
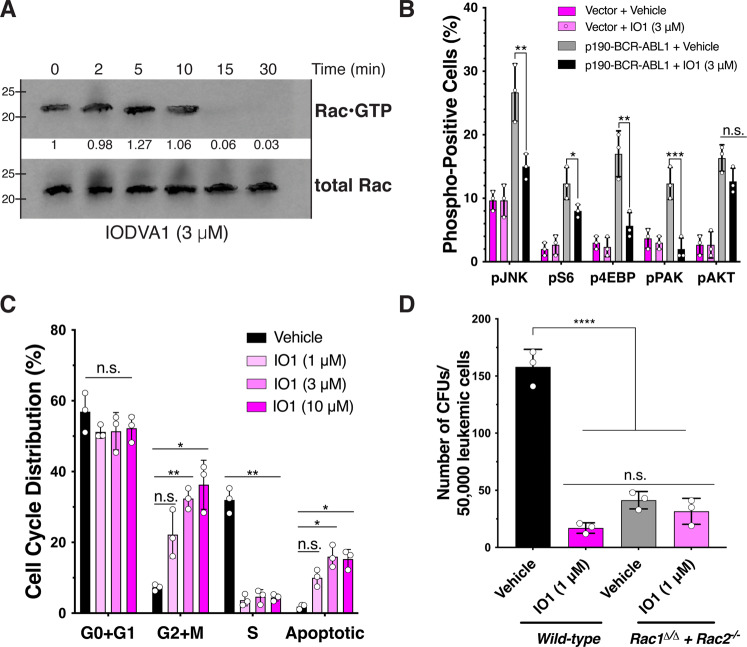
IODVA1 targets RAC activation and signaling. **A** Effect on active RAC (RAC•GTP) in p190-BCR-ABL1-expressing Ba/F3 cells treated with IODVA1 (3 μM) as indicated. Active RAC levels were assessed by pulldown using GST-PAK-GBD, followed by immunoblotting and densitometric quantification. Results are representative of two independent experiments. **B** Bar graph of results of the phospho-flow cytometry analysis of pJNK, pS6, p4EBP, pPAK1, and pAKT of Ba/F3 cells expressing empty vector (dark and light lilac) or p190-BCR-ABL1 (gray and black) and treated with vehicle control or IODVA1 (3 μM) for 30 min. **C** Bar graph of a representative cell-cycle analysis of p190-BCR-ABL1-Ba/F3 cells treated with vehicle control or IODVA1 (1, 3, and 10 μM) for 20 h. **D** Quantification of the average number of colonies of bone marrow wild-type and *Rac1*^*Δ/Δ*^*+Rac2*^*-/-*^ p190-BCR-ABL1 leukemic cells treated with vehicle control or IODVA1 (IO1, 1 μM). ns not significant, *p ≤ 0.05, **p ≤ 0.01, ***p ≤ 0.001, ****p ≤ 0.0001 using one- (panels **B** and **D**) or two-way ANOVA (panel **C**) with Tukey’s multiple comparison test.

As RAC is activated downstream of BCR-ABL1, we tested if IODVA1 inhibits RAC activation in BCR-ABL1-transformed cells. IODVA1 (3 μM) treatment substantially decreased levels of active RAC within 10–15 min (Fig. 3A). IODVA1 is specific to RAC (IC50 = 1 μM) and is less effective on CDC42 and not at all on RHOA (Supplementary Fig. S4A, B), consistent with our previous observations in MDA-MB-231 cells [11]. As a result of decrease in activation of RAC, phosphorylation levels of downstream signaling molecules were decreased—JNK by 1.8-fold (*p* = 0.004), S6 by 1.5 (*p* = 0.046), 4EBP by 3.0 (*p* = 0.001), and PAK by 6.1 (*p* = 0.0005) fold, respectively. Importantly, IODVA1-induced decrease in effector phosphorylation levels is specific to BCR-ABL1-Ba/F3. IODVA1 did not affect the phosphorylation levels of AKT regardless of the BCR-ABL1 status (Fig. 3B).

Significantly, IODVA1 decreases phospho-levels of RAC downstream pro-survival PAK and decreases inhibitory phosphorylation of pro-apoptotic BAD Ser136 activities within minutes of cell exposure (Supplementary Fig. S4C). The decrease in PAK and BAD phosphorylation suggests that IODVA1 promotes reduction in survival and induction of apoptosis. To further test this hypothesis, we analyzed the cell cycle of p190-BCR-ABL1 leukemic progenitor cells (EGFP^+^/B220^dim^) treated with vehicle control or IODVA1 (1–10 μM) (Fig. 3C). IODVA1 did not affect the G0+G1 phase but significantly changed the distribution of the G2+M, S, and apoptotic phases. It increased the percentage of cells in the G2+M phases, reduced the S-phase 8-fold (*p* = 0.005) and increased apoptosis by at least 5.3 times. Therefore, IODVA1 increases percentage of cells in G2/M phase, induces S-phase arrest, and increases apoptosis.

### RAC-deficient cells do not respond to IODVA1

To confirm that IODVA1 targets RAC-dependent pathways, we assessed its effects in a *Rac2*-null background. *Rac1*^*Δ/Δ*^*+Rac2*^*-/-*^ murine cells, which show severe reduction in RAC1 expression and are deficient in RAC2 (Supplementary Fig. S4D), and wild-type cells were transduced with p190-BCR-ABL1 [21] and tested for colony-forming ability in the presence of IODVA1 (Fig. 3D). IODVA1 significantly inhibited colony-forming ability of wild-type but not *Rac1*^*Δ/Δ*^*+Rac2*^*-/-*^ leukemic cells, suggesting that they are insensitive to IODVA1. *Rac1*^*Δ/Δ*^*+Rac2*^*-/-*^ leukemic cells treated with vehicle or IODVA1 formed 2.4 times more colonies than wild-type leukemic cells treated with IODVA1. Combined with the biochemical data, these data support the idea that IODVA1 targets RAC activity in leukemic models and inhibits its downstream pro-survival signals.

### IODVA1 inhibits the RacGEF VAV3

**Fig. 4:**
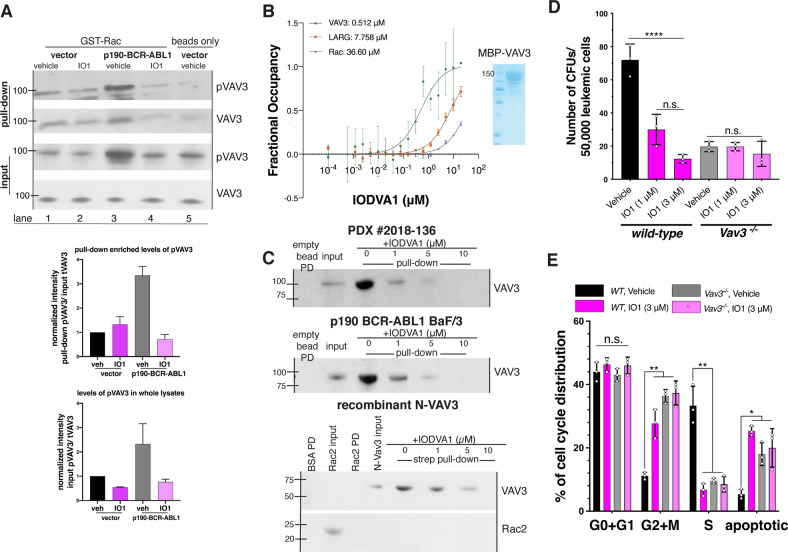
IODVA1 targets VAV3 in vitro and in vivo. **A** Results of GST-RAC pulldown from empty vector- and p190-BCR-ABL1-Ba/F3 cells treated with vehicle control or IODVA1 (IO1, 3 μM) for 30 min. Bead-bound protein complexes were washed, separated on SDS-PAGE, and immunoblotted for phospho- and total VAV3 and quantified. Input total (VAV3) and phospho (pVAV3) were used as reference. Quantification against total VAV3 is shown. Results are representative of at least two independent experiments. **B** Binding affinity (K_d_) between IODVA1 and VAV3 (green), LARG (brown), or RAC·GDP (blue). The microscale thermophoresis signal expressed as fractional occupancy was plotted against IODVA1 (0.1 nM–20 μM) and fitted to yield K_d_. Results are shown as mean ± SD of three independent experiments. **C** Results of biotinylated IODVA1 pulldown from PDX Ph^+^ B-ALL 2018-136 and p190-BCR-ABL1-Ba/F3 cell lysates and from recombinant N-VAV3. Neutravidin bead-bound protein complexes were washed, separated on SDS-PAGE, and immunoblotted for VAV3 and quantified. Results are representative of at least two independent experiments. **D** Quantification of the average number of colonies made by bone marrow wild-type and *Vav3*^*-/-*^ p190-BCR-ABL1 leukemic cells treated with vehicle control or IODVA1 (IO1, 1 and 3 μM). Experiments were performed in triplicates. **E** Bar graph summarizing representative cell-cycle analysis experiment of wild-type (black and dark lilac bars) and *Vav3*^*-/-*^ (gray and light lilac bars) p190-BCR-ABL1-bone marrow cells and treated with vehicle control or IODVA1 (IO1, 3 μM) for 20 h, performed in triplicates. n.s. not significant, *p ≤ 0.05; **p ≤ 0.01; ****p ≤ 0.0001 based on one- (**D**) or two-way (**E**) ANOVA with Tukey’s multiple comparisons test.

RAC activation and signaling is tightly regulated; thus, we argued that the effects on RAC are caused by IODVA1 targeting one RAC regulator. Our biochemical assays show that IODVA1 does not stimulate the activity of the RAC negative regulators p50GAP and RhoGDI1 (Supplementary Fig. S4E–G), so we turned to positive regulators GEFs and posited that IODVA1 inhibits one RAC-specific GEF leading to its inactivation. While several RacGEFs have been associated with leukemogenesis [22–28], VAV3 was shown to play a central role [12]. We thus focused on VAV3 and tested if IODVA1 inhibits VAV3 binding to RAC. p190-BCR-ABL1 or empty vector-expressing Ba/F3 were treated with IODVA1 (3 μM) or vehicle control, subjected to GST-RAC pulldown and immunoblotted for pVAV3 and VAV3 (Fig. 4A). There was no significant change in levels of VAV3 or pVAV3 bound to RAC in empty vector-expressing Ba/F3 cells regardless of the treatment (Fig. 4A, lanes 1 and 2). Consistent with previous studies [12], expressing p190-BCR-ABL1 increased levels of pVAV3 by 3.2-fold in vehicle-treated cells and binding between pVAV3 and RAC (Fig. 4A, input lane 3 and pulldown lane 3). IODVA1 treatment of cells expressing p190-BCR-ABL1 decreased levels of overall pVAV3 by 4-fold (Fig. 4A, input lane 4). This decrease is accompanied by a reduced binding of pVAV3/VAV3 to RAC (Fig. 4A, pulldown lane 4). Thus, our data suggest that IODVA1 inhibits overall VAV3 activation leading to a decreased binding to and activation of RAC in BCR-ABL1-expressing cells.

We then measured the binding affinity (K_d_) of IODVA1 to recombinant VAV3 and RAC1 using microscale thermophoresis (MST) with catalytic domain (DH/PH) of RhoGEF LARG serving as negative control. IODVA1 binds to VAV3 in a 1:1 molar ratio with a K_d_ of 512 nM (Fig. 4B). The MST signal for RAC1 and LARG showed no saturation at the highest IODVA1 concentration tested, suggesting no meaningful interaction.

We further confirmed that IODVA1 binds to VAV3 by incubating a biotinylated analog of IODVA1 with lysates from Ph^+^ B-ALL patient 2018-136 or from p190-BCR-ABL1-expressing Ba/F3 cells and performing an avidin pulldown assay (Fig. 4C). Both cell lysates contained detectable levels of VAV3 as detected by immunoblotting that was concentrated 5–7 folds on biotinylated IODVA1 while the empty beads did not bind any VAV3 (lanes 1–3). Free IODVA1 (1–10 μM) was able to compete VAV3 binding to the biotinylated immobilized IODVA1 in a dose-dependent manner (lanes 4–6). Next, we repeated the same experiment but with recombinant RAC2 and N-VAV3 lacking the C-terminus SRC-homology domains. Biotinylated IODVA1 but not empty beads pulled N-VAV3 but not RAC2 and the addition of IODVA1 dissociated N-VAV3 from the conjugated beads (Fig. 4C). Together, these data further confirm that IODVA1 binds to VAV3 and that the C-terminus SH2/SH3 adaptor domain is not required for binding.

### VAV3-deficient leukemic cells do not respond to IODVA1 in vitro and in vivo

To further validate VAV3 as target of IODVA1 and to test its specificity in our leukemic model, we studied the effects of IODVA1 on leukemic cells from Vav3 knockout (*Vav3*^*-/-*^) mice [1, 12]. We argued that if IODVA1 targets VAV3, then *Vav3*^*-/-*^ cells should be less sensitive to IODVA1. Wild-type or *Vav3*^*-/-*^ murine BM leukemic cells expressing p190-BCR-ABL1 (EGFP^+^/B220^+^) were subjected to colony-forming assay in the presence of IODVA1 (Fig. 4D). The number of colonies formed by wild-type leukemic cells expressing Vav3 (mean ± SD of 72 ± 9.5) decreased on average by 2.4- and 7-fold in the presence of 1 and 3 μM IODVA1 (30 ± 9.2 and 12 ± 2.5), respectively. *Vav3*^*-/-*^ leukemic BM cells on the other hand formed similar number of colonies when grown in the presence of vehicle control (20 ± 3.1) or IODVA1 (20 ± 2.5 and 15 ± 7.5) suggesting they lost sensitivity to our drug. We did not observe significant difference in the number of colonies formed by *Vav3*^*-*/-^ leukemic cells and by IODVA1-treated wild-type leukemic cells. Similarly, cell-cycle analysis shows that *Vav3*^*-/-*^ cells expressing p190-BCR-ABL1 were not affected by IODVA1 (Fig. 4E). Taken together, our data suggest specificity of IODVA1 to VAV3 as wild-type leukemic cells respond to IODVA1, while *Vav3*^*-/-*^ leukemic cells are irresponsive and mimic IODVA1-treated wild-type Vav3 leukemic cells.

**Fig. 5:**
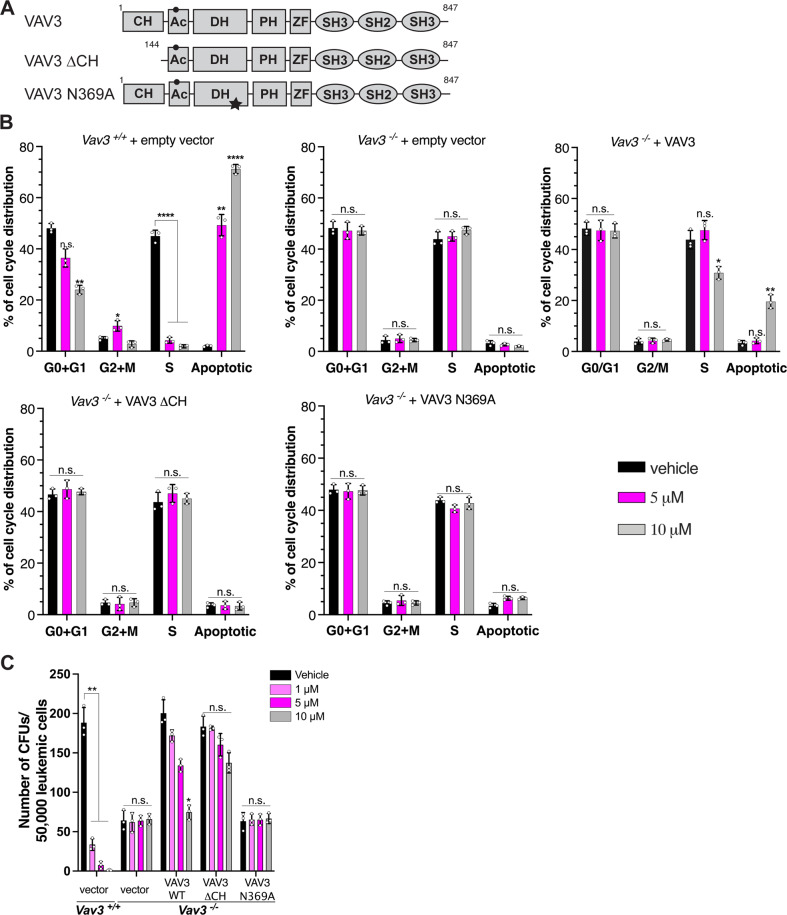
Expression of transgenic VAV3 re-sensitizes *Vav3*-deficient cells to IODVA1. **A** Schematic of domain architecture of full-length, ΔCH, and GEF-mutant VAV3; calponin homology (CH), acidic region (Ac), Dbl-homology (DH), pleckstrin-homology (PH), zinc finger (ZF), SRC-homology 2 and 3 (SH2/SH3). Black circle and star indicate Y173 and N369, respectively. **B** Bar graphs of representative cell-cycle analysis experiment of wild-type (*Vav3*^*+/+*^) and Vav3-deficient (*Vav3*^*-/-*^) p190-BCR-ABL1 leukemic bone marrow cells expressing empty vector, full-length VAV3, ΔCH, or N369A mutants and treated with vehicle control (black) or IODVA1 at 5 (lilac) or 10 μM (gray) for 18 h. **C** Quantification of the average number of colonies made by wild-type and *Vav3*^*-/-*^ p190-BCR-ABL1 leukemic bone marrow cells expressing empty vector, or the VAV3 constructs used in (**B**) and treated with vehicle control (black) or IODVA1 at 1 (light lilac), 5 (dark lilac), and 10 μM (gray). n.s. not significant, *p ≤ 0.05; **p ≤ 0.01; ****p ≤ 0.0001 based on two-way ANOVA with Tukey’s multiple comparison test.

Next, we reasoned that if IODVA1 targets VAV3, rescuing *Vav3*^*-/-*^ leukemic cells by expressing exogenous VAV3 should re-sensitize those cells to IODVA1. We expressed full-length VAV3, the dominant active ΔCH mutant or the N369A exchange-deficient mutant [29] in *Vav3*^*-/-*^ p190-BCR-ABL1-transformed murine BM leukemic cells and analyzed cell-cycle changes post vehicle or IODVA1 treatment (Fig. 5A, B). Full-length VAV3 but not the empty vector re-sensitized the *Vav3*^*-/-*^ BM leukemic cells to IODVA1 as shown by a 5.8-fold increase in apoptosis (*p* = 0.008) and a 15% decrease (*p* = 0.02) in cells in the S-phase at 10 μM. The ΔCH or the GEF-activity deficient mutants did not re-sensitize *Vav3*^*-/-*^ leukemic cells to IODVA1 even at the highest concentration. In addition, we performed the rescue experiments using the colony-forming assay in the presence of IODVA1 (1, 5, and 10 μM) or vehicle control (Fig. 5C). Re-introducing full-length or ΔCH VAV3 results in similar number of colonies as with wild-type BM leukemic cells (black bars, mean of 200.3 and 183.3 vs 188.3, respectively), a 3-fold increase from *Vav3*^*-/-*^ leukemic cells expressing the empty vector (mean = 64.3 colonies). Re-introducing the GEF-deficient mutant has no effect on the colony formation ability of *Vav3*^*-/-*^ leukemic cells (mean = 63.3 colonies) consistent with the importance of sustained Rac activity for proliferation of leukemic cells. Importantly, *Vav3*^*-/-*^ leukemic cells expressing VAV3 respond to IODVA1 in a dose-dependent manner. At 10 μM IODVA1, the number of colonies made by *Vav3*^*-/-*^ cells expressing full-length VAV3 is reduced by a third and becomes like that made by *Vav3*^*-/-*^ expressing empty vector (gray bars, 75 vs 66 colonies). On the other hand, ΔCH and GEF-deficient mutants expressing leukemic cells are not sensitive to IODVA1. Taken together, the cell-cycle and proliferation experiments show that exogenous expression of VAV3 in *Vav3*^*-/-*^ leukemic cells increases their dependency on VAV3 GEF-dependent proliferative pathways and re-sensitizes them to IODVA1. The reduced sensitivity of the ΔCH mutant to IODVA1 suggests that this domain is part of its binding site on VAV3.

Next, we tested if the lack of response to IODVA1 by *Vav3*^*-/-*^ cells holds in vivo. We used wild-type or *Vav3*^*-/-*^ LDBM cells in p190-BCR-ABL1 transplantation model and treated mice as before. IODVA1 did not have any effect on the survival of *Vav3*^*-/-*^ leukemic mice supporting the hypothesis that VAV3 is IODVA1’s target in vivo (Supplementary Fig. S5A). The persistency of leukemia in vivo in *Vav3*^*-/-*^ chimeras suggests that *Vav3*^*-/-*^ BCR-ABL1 leukemia has evolved mechanisms of escape relying on RAC-independent pathways such as STAT3 signaling pathways (Supplementary Fig. S5B–D).

Ba/F3 cells that exogenously express full-length or dominant active ΔCH VAV3 in the p190-BCR-ABL1 but not in the empty vector background show increased resistance to IODVA1 (Supplementary Fig. S5E). To test IODVA1’s specificity, we repeated the pulldown assays with biotinylated IODVA1 (Fig. 4C) and checked by immunoblots if the RacGEFs VAV1 or PREX1 are present in the protein complexes on the neutravidin beads. We argued that if IODVA1 binds other proteins, VAV3 closest homologs, e.g., VAV1, are likely candidates. PREX1 was chosen because, like VAV-proteins, its activity is PIP3 regulated. Whereas VAV3 was concentrated on biotinylated IODVA1 beads, VAV1 and PREX1 were not even though they were clearly present in the p190-Ba/F3 and PDX 2018-136 cell lysates (Supplementary Fig. S5F). Together, our data show that *Vav3*-deficient leukemia progenitor cells do not respond to IODVA1 in cellular and in vivo assays while re-introducing VAV3 re-sensitizes them to IODVA1. The weaker response of the VAV3 ΔCH mutant to IODVA1 suggests that the calponin homology domain regulates IODVA1’s activity. Our data are consistent with the idea that VAV3 is IODVA1’s target in vivo and in vitro.

### IODVA1 decreases the VAV3/RAC signaling axis in PDX models

**Fig. 6:**
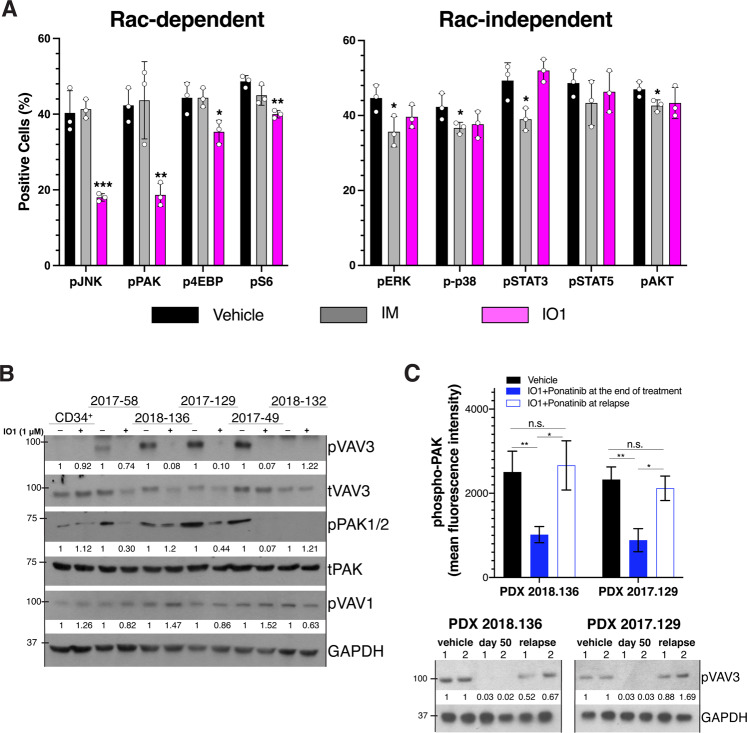
IODVA1 decreases the VAV3/RAC signaling pathway in vivo. **A** Bar graphs of pharmacodynamic assessment of leukemic progenitor cells (%) from the 2-week treated mice with vehicle control (black), imatinib (gray), or IODVA1 (lilac) from Fig. 2A using phospho-flow cytometry of the indicated RAC-dependent and -independent effectors. *p ≤ 0.05, **p ≤ 0.01, ***p ≤ 0.001 using one-way ANOVA with Dunnet’s multiple comparison test. **B** CD34^+^ cells or anti-human CD19^+^/CD45^dim^ sorted cells from the indicated PDX specimen (see Supplementary Table S1) were incubated in the presence of vehicle or IODVA1 (1 μM) for 30 min and processed for immunoblotting analysis for phospho- and total VAV3, -PAK1, and phospho-VAV1. Phospho-proteins band densities were normalized to the total protein (pVAV3 and pPAK) or GAPDH (pVAV1) and quantified relative to their respective vehicle treatments. **C** Bone marrow aspirates from vehicle-treated mice at time of death (day 32) or IODVA1 + ponatinib-treated mice from PDX 2018-136 and PDX 2017-129 at the end of treatment (day 50) and the last day post-relapse from Fig. 2C, D were subjected to phospho-PAK1 analysis by flow cytometry (bar graph) or resolved by SDS-PAGE, blotted for pVAV3, and normalized to GAPDH (immunoblot). Mice were randomly pooled into two groups of three labeled 1 and 2. n.s. not significant, *p ≤ 0.05; **p ≤ 0.01 based on one-way ANOVA with Tukey’s multiple comparisons test.

To confirm that IODVA1 targets VAV3/RAC signaling in vivo, EGFP^+^/B220^+^ LDBM cells were isolated from the 2-week treated p210(T315I) leukemic mice (Fig. 2A) and analyzed for phosphorylation status of pro-proliferative RAC-dependent effectors JNK, PAK1/2/3, 4EBP, and S6 and the RAC-independent effectors ERK1/2, STAT3, STAT5, p38, and AKT. IODVA1 resulted in significant decreases in pJNK by 55% (*p* = 0.003), pPAK1 by 56% (*p* = 0.008), p4EBP by 20.3% (*p* = 0.026), and pS6 by 17.8% (*p* = 0.002), respectively (Fig. 6A). Phosphorylation levels of ERK, p38, STAT3, STAT5, and AKT were not affected by IODVA1. Interestingly, imatinib had the opposite effect. The decrease in PAK1, JNK, S6, and 4EBP activity in LDBM cells from IODVA1-treated p210(T315I) mice in pharmacodynamics studies mirrors the decrease observed in IODVA1-treated Ba/F3 cells (Fig. 3C). Taken together, IODVA1 overcomes TKI resistance and eliminates TKI-resistant leukemic stem/progenitor cells in vivo likely by inhibiting VAV3/RAC downstream signaling pathways.

The responses of PDX cells to IODVA1 ex vivo (Supplementary Fig. S3A–C) correlate with VAV3 status—levels of pVAV3 were high in Ph^+^ PDX 2018-136 and 2017-129 cells and Ph-like PDX 2017-49 cells and decreased by 90% following IODVA1 treatment (Fig. 6B). Human cord blood CD34^+^ cells were used as negative control as they were not sensitive to IODVA1 and do not have high levels of pVAV3. Levels of pVAV3 and pPAK1 were not detectable in weakly responsive Ph^+^ PDX cells 2017-58 and Ph-like 2018-132 cells regardless of the treatment. pVAV1 levels on the other hand were unchanged by IODVA1 in any of the patient samples tested. Similarly, the pVAV3/VAV3 levels of Ph-like PDX 2016-79 and 2018-132 cells with the same (IGH-CLRF2; JAK2) mutations correlate with their response to IODVA1 (Supplementary Fig. S3B, D) and are consistent with levels of pVAV3/VAV3 being a biomarker for IODVA1 response.

To further confirm the effect on VAV3/RAC/PAK pathway in the 2018-136 and 2017-129 PDX models, we checked levels of pVAV3 and pPAK1/2 in BM cell aspirates of vehicle and IODVA1 + ponatinib-treated mice from the two PDX models (Fig. 6C). pVAV3 levels are high in vehicle-treated mice at the time of death but pVAV3 was hardly detectable in IODVA1 + ponatinib-treated mice at the end of treatment (day 50). Ending the combo treatment increased pVAV3 levels as a pVAV3 band is obvious at the time of animal sacrifice, day 92 or 78, respectively. pPAK1/2 follows the same pattern.

The observation that IODVA1 is superior to dasatinib (Fig. 2C–D) prompted us to check the ability of the ABL1-TKI to inhibit VAV3 phosphorylation. We argued that dasatinib, an inhibitor of BCR-ABL1 and SRC-kinase family involved in VAV3 activation, should inhibit VAV3. We compared levels of pVAV3 in BM of PDX 2018-136 mice treated with dasatinib or IODVA1 (Supplementary Fig. S5G). IODVA1, but not dasatinib significantly decreased VAV3 activation. Thus, the effect of IODVA1 on prolonging the survival of PDX mice correlates with its superior ability at inhibiting VAV3 activation.

## Discussion

Ph^+^ and Ph-like B-ALL confer a much poorer prognosis compared to B-ALL with other cytogenetic or molecular abnormalities [16, 17, 30]. Best therapeutic approaches combining chemotherapy, ABL1-TKIs, and immunotherapy proved to be only partly effective in Ph^+^ B-ALL patients [16]. The relative failure of TKI therapy is due, among others, to development of resistance-inducing mutations, such as ABL1-T315I and to recurrent somatic alterations in key signaling pathways such as the RAS/ERK, PI3K/AKT, B-cell development and differentiation, RAC/PAK, and Janus kinases [31, 32]. IODVA1 has superior potency against pediatric patient samples with Ph^+^ B-ALL including TKI-resistant BCR-ABL1(T315I) than currently administered therapies. Its success in patient samples of Ph-like and MLL rearrangements (Supplementary Fig. S3) illustrates its effect in a variety of tyrosine kinase receptors. Thus, IODVA1 is well suited to treat Ph^+^ and Ph-like and probably other leukemias with a superior suppression of growth and survival.

VAV3 is a multi-domain tyrosine phosphorylation–dependent RacGEF that functions downstream of several different signaling molecules including immune response receptors, G-protein-coupled receptors, protein tyrosine kinases, and integrins and is a critical component of BCR-ABL1-induced RAC activation [12, 33]. Thus far, no small-molecule inhibitor of VAV3 has been reported. If the small-molecule EHop-016 was shown to inhibit the VAV1/RAC1 interaction, it is not clear if it targets VAV1 directly [24]. Our data show that IODVA1 binds tightly to VAV3 (K_d_ = 512 nM, Fig. 4B, C), prevents its activation, and the activation of RAC in vitro and in vivo. In accordance with this mechanism of action, IODVA1 significantly decreases the survival of genetically diverse PDX B-ALL cells in a VAV3-dependent manner (Supplementary Fig. S3 and Fig. 6B). One can argue that IODVA1 inhibits BCR-ABL1 or the transmembrane or cytosolic protein tyrosine kinases responsible for VAV3 phosphorylation [33]; however, this is unlikely since IODVA1 is not a kinase inhibitor [11] and BCR-ABL1 phosphorylation levels or activity are not affected by IODVA1 (Supplementary Fig. S2). IODVA1 targeting of VAV3 seems specific as it has no effect on *Vav3*^*-/-*^ cells in vitro or in vivo. The possibility that IODVA1 inhibits the homologous VAV1/2 exists but based on the loss of IODVA1’s efficacy in *Vav3*^*-/-*^ models (Fig. 4C–E), its inability to affect VAV1 phosphorylation levels in primary patient cells (Fig. 6B), or its inability to interact with VAV1 (Supplementary Fig. S5F), this inhibition is unlikely. Significantly, IODVA1 has no effect on *Vav3*^*-/-*^ murine BM leukemic cells and does not affect the survival of a mouse model of *Vav3*^*-/-*^ p190-BCR-ABL1-induced leukemia. In addition, IODVA1 has no effect on *Rac1*^*Δ/Δ*^*+Rac2*^*-/-*^ leukemic cells (Fig. 3D). These in vivo and in vitro results suggest that off-target effect on another RacGEF is unlikely, as increases in mouse survival or cell proliferation were not detected.

Our in vitro cell cycle and colony formation data indicate that IODVA1 phenocopies genetic deletion of VAV3 (Fig. 4D, E), consistent with no off-target effects. The IODVA1-triggered increase in apoptosis (Figs. 3C and 4E) is also in line with the increase in apoptosis observed in *Vav3*^*-/-*^ leukemic cells [12]. Results from the colony formation assays with the *Rac1*^*Δ/Δ*^*+Rac2*^*-/-*^ leukemic cells (Fig. 3D) are also consistent with this idea. The colony formation assays (Figs. 3D and 4D) and the VAV3 rescue experiments (Fig. 5C) point to the importance of the VAV3/RAC pathway in the pathophysiology of Ph^+^ leukemias. We noticed, however, that the *Rac1*^*Δ/Δ*^*+Rac2*^*-/-*^ leukemic cells resulted in higher number of colonies than cells treated with IODVA1. This can be explained by residual Rac1 (Supplementary Fig. S4D), which is pro cell proliferation or by the RAC-independent pathways regulated by VAV3 that are not inhibited by IODVA1. Likewise, there is a noticeable difference between IODVA1’s pharmacological action in vivo, which increases survival of leukemic mice, and VAV3 genetic deletion, which results in premature leukemic mice death (Supplementary Fig. S5A). Our phospho-flow cytometric data show, however, that in the background of *Vav3*^*-/-*^, BCR-ABL1 leukemic cells increase AKT and STAT3 pathway signaling (Supplementary Fig. S5C, D), which may be the mechanism of adaptation responsible for LSC proliferation and death of *Vav3*^*-/-*^ leukemic mice (see also [12]).

VAV3 can be roughly divided into two halves: (1) a RAC-binding N-terminal half and a (2) C-terminal adaptor module (Fig. 5A). Cell stimulation releases autoinhibition by the N-terminal calponin homology CH-domain and acidic stretch (Ac) and the C-terminal SH3 domain via phosphorylation of conserved Tyr-residues (e.g., Tyr173), thus allowing GTPase access to the DH domain [34]. Based on our biochemical data (Figs. 4A, B and 6B and Supplementary Fig. S6B), we argue that IODVA1 inhibits VAV3 phosphorylation by preventing the access of tyrosine residues in the Ac-stretch to SRC kinases. We postulate that IODVA1 likely locks VAV3 into the autoinhibitory state, thus preventing RAC from accessing the DH domain for activation. Consistent with this picture are our rescue data with the ΔCH mutant of VAV3, which unlike full-length VAV3, lost its sensitivity to IODVA1 (Fig. 5B). These data suggest that the VAV3 calponin homology domain is part of IODVA1 binding site. The pulldown with biotinylated IODVA1 (Fig. 4C) suggests that the C-terminal SH2/SH3 domains are dispensable for IODVA1 binding. Dissection of the VAV3/IODVA1 interaction and precise mapping of the binding site are currently underway.

In conclusion, we have shown that pharmacological inhibition of VAV3 by IODVA1 in preclinical models is therapeutically superior to inhibiting upstream kinases and thus an attractive therapeutic strategy to treat Ph^+^ and TKI-resistant Ph^+^ B-ALL. This strategy should benefit other malignancies where VAV3 is a target. We thus expect IODVA1 to have a broader therapeutic application. In addition, IODVA1 constitutes an exceptional tool to dissect the VAV3/RAC signaling axis. Broadly, RhoGEFs are multi-domain proteins that are regulated by autoinhibition. Consequently, small molecules that stabilize the autoinhibited conformation of RhoGEFs and inhibit their activity could be developed into drugs to treat human cancers.

## Methods

Reagents, antibodies, plasmids, recombinant protein cloning, expression and purification, SDS-PAGE, pulldown assays and immunoblotting, MST, cell lines, virus production, transduction, transplantation, ex vivo drug treatment, flow cytometry, histology, colony formation assays, and cell-cycle analysis are described in the Supplementary Methods section.

### Animals and in vivo drug administration

*Vav3*-deficient mice [35] and *Rac1*^*Δ/Δ*^*+Rac2*-deficient [1] mice have been described previously. C57Bl/10 (females, 8–16 weeks old) and NSG (NOD/SCID/IL2RG^-/-^ males and females, 8–14 weeks old) mice (The Jackson Laboratory, Bar Harbor, ME and Harlan, Indianapolis, IN) were used as donors and/or recipients of transduction/transplantation models. Animals were maintained at an Association for Assessment and Accreditation of Laboratory Animal Care–accredited, specific-pathogen-free animal facility under Cincinnati Children’s Hospital Medical Center Institutional Animal Care and Use Committee–approved protocol. For in vivo drug administration, Alzet implantable osmotic pumps (Durect, Cupertino, CA) were used according to the manufacturer’s protocol and implantation was done as described previously [1]. To determine the lowest IODVA1 dose required to achieve mouse survival, pumps with 0.25 and 0.5 mM IODVA1 were used in the in vivo models of p190-BCR-ABL1-expressing LDBM cells. For the therapy-resistant multi-lesion PDX models treatment, pumps with IODVA1 at 4 mM were used to maximize survival.

### Clinical samples

Clinical samples were obtained from patients at CCHMC according to Institutional Review Board Approved protocols (#2008-0021 and #2008-0658). Post RBC lysis, isolated WBCs were mixed with α-CD3 antibody to eliminate xenogenic graft-versus-host disease potential prior to injection into busulfan-conditioned NSG or NRG mice [36].

For in vivo drug treatments, cells isolated from PDX 2018-136 and 2017-129 were transplanted into sublethally irradiated (2.75 Gy) NSG mice and treated as described above in “Animals and in vivo drug administration” and in Supplementary Data.

## Supplementary information

Supplementary Data
