# Supplementary material for: Inhibition of the RacGEF VAV3 by the small molecule IODVA1 impedes RAC signaling and overcomes resistance to tyrosine kinase inhibition in acute lymphoblastic leukemia
Source: Leukemia. 2021 Oct 28;36(3):637–47. doi: 10.1038/s41375-021-01455-3 (PMC8885421; doi:10.1038/s41375-021-01455-3)
Supplement: Supplementary file 1 — Supplementary Data [file 41375_2021_1455_MOESM1_ESM.pdf]

Supplemental Data

Inhibition of the RAC Activator VAV3 by the Small Molecule IODVA1 Impedes RAC Signaling and Overcomes Resistance to Tyrosine Kinase Inhibition in Acute Lymphoblastic Leukemia

Shailaja Hegde [1,2], Anjelika Gasilina [1], Mark Wunderlich [1], Yuan Lin [1], Marcel Buchholzer [3], Oliver H.F. Krumbach [3], Mohammad Akbarzadeh [3], Mohammad Reza Ahmadian [3], William Seibel [4], Yi Zheng [1,5], John P. Perentesis [4], Benjamin Mizukawa [1], Lisa Privette Vinnedge [4,5], José A. Cancelas [1,2,5], and Nicolas N. Nassar [1,5*]

[1]: Division of Experimental Hematology and Cancer Biology, Children's Hospital Research Foundation, 3333 Burnet Ave, Cincinnati, OH 45229

[2]: Hoxworth Blood Center, University of Cincinnati Academic Health Center, OH, USA

[3]: Institute of Biochemistry and Molecular Biology II, Medical Faculty, Heinrich-Heine-University, Düsseldorf 40225, Germany

[4]: Division of Oncology, Cincinnati Children's Hospital Medical Center, Cancer and Blood Diseases Institute, 3333 Burnet Ave, Cincinnati, OH 45229

[5]: Department of Pediatrics, University of Cincinnati College of Medicine, 3230 Eden Ave, Cincinnati, OH 45267

*: To whom correspondence should be addressed at nicolas.nassar@cchmc.org

Disclosure of Potential Conflicts of Interest: No potential conflicts of interest are disclosed by the authors.

Supplemental Results

IODVA1 specifically targets BCR-ABL1 B-ALL cells in vitro. We tested the efficacy of IODVA1 in two commonly used Ph^+ cell models, Ba/F3 cells transduced with p190- or p210-BCR-ABL1 and NALM-1 cells (1). NALM-1 are human lymphoblastic cells that express p190-BCR-ABL1 but not *Ikaros* (IKZF1) (2). Cells were grown in suspension in the presence of IODVA1 and counted daily for 3 days by trypan blue exclusion. At 3 μM, IODVA1 reduced the viability of p190- and p210-BCR-ABL1 expressing cells by 75 ± 4% (SEM, N = 9) at day 1; viability of Mieg3 empty vector expressing cells was not affected (**Fig. S1A**). Plot of the concentration-dependent cell survival at the 72-hour time point shows that IODVA1 inhibits survival of p190-BCR-ABL1-Ba/F3 cells grown in the presence of IL-3 with an EC_{50} of 380 nM (95% CI 302 to 473 nM). IL-3 withdrawal renders p190-BCR-ABL1 Ba/F3 cells more sensitive to IODVA1 (EC50 of 9 nM). Survival of empty vector expressing Ba/F3 cells was not affected by IODVA1 up to 5 μM and was reduced by 25.5% at 20 μM (**Fig. S1B**). Similarly, IODVA1 decreases survival of NALM-1 cells with EC50 of 677 nM (95% CI 600 to 767.5 nM).

To test if the action of IODVA1 is reversible, we washed out IODVA1 post-24-hour incubation and counted cells for 6 additional days (**Fig. S1C**). As expected, death of p190-BCR-ABL1 Ba/F3 cells occurred following IODVA1 incubation and persisted even after IODVA1 removal (washout, arrow). By day 3, allowing for one round of cell division, all cells were dead, suggesting that IODVA1 triggers an irreversible cell death program.

We tested the ability of IODVA1 to inhibit the clonogenic ability of BCR-ABL1 expressing Ba/F3 cells in soft agar. At 10 μM, IODVA1 completely abolished colony formation of Ba/F3 cells transduced with p190-BCR-ABL1 vector (**Fig. S1D**). Colonies

are noticeable at 1 μM albeit they are smaller than vehicle DMSO control, indicative of slowed cell division rate.

IODVA1 decreases in vivo leukemic burden and prevents leukemia-related death. To assess the effect of IODVA1 on leukemia burden, we performed flow cytometric analysis on $EGFP^+/B220^+$ progenitors from peripheral blood of vehicle- and drug-treated mice. All groups had comparable levels of circulating blasts between 12 and 16% at the start of the treatment (**Fig. S1E**). As the treatment progressed, the number of $EGFP^+/B220^+$ cells decreased to reach 1 to 2% at week 4. Although the data consistently showed a trend for the IODVA1+imatinib combination to eliminate leukemic burden better than either drug alone, analysis revealed no statistical significance ($p = 0.186$).

IODVA1 eradicates leukemic propagating activity. At the 3- and 5-week time points, leukemic progenitor cells ($EGFP^+/B220^+$) from the peripheral blood (PB) of secondary transplanted mice from **Fig. 1D** were analyzed by flow cytometry and plotted as percentage of total PB for the 10^6-cell dilution. **Fig. S1F** shows that unlike imatinib-treated mice where $EGFP^+/B220^+$ cells constituted 23.2 ± 5.4 % of B-cells 5 weeks post transplantation, IODVA1- and IODVA1+imatinib-treated mice had a 4.2 ± 0.7 and 3.0 ± 0.3%, respectively, $EGFP^+/B220^+$ leukemic B-cells at the same time-point. Similar trends in mice survival and leukemic burden were obtained for the higher dilutions (**Fig. S1G - S1J).** We have previously evaluated IODVA1 for any hematological toxicity and detected no adverse changes in body weight or hematological parameters (3). Together, these data indicate that IODVA1 eradicates all leukemogenic cells *in vivo*, including the ones responsible for

propagation and possibly relapse and that IODVA1 is more efficient than imatinib at decreasing the leukemic cell burden.

IODVA1 decreases survival of patient-derived leukemia cells. Cells from PDX models representing pediatric Ph^+, Ph-like with a diverse series of genetic aberrations, and a few cases of MLL-rearranged B-ALL patients (**Table S1)** generally responded positively to IODVA1 *ex vivo* (**Fig. S3)**.

Ph^+ B-ALL. Cells from patient #2017-58 with a dual Ph^+ (BCR-ABL1) and Ph-like (P2RY8-CRLF2) rearrangement were treated with ABL1-TKI dasatinib, JAK inhibitor ruxolitinib, dasatinib and ruxolitinib combination (das + rux), CDK-inhibitor abemaciclib, or IODVA1 (**Fig. S3A,** left panel**)**. These cells clearly responded to dasatinib, ruxolitinib, and the combination. IODVA1 was not as potent as it decreased their proliferation by only 40% at 1 μM and had no effect at 0.2 μM. Cells from relapsed patient #2018-136 with Ph^+ (BCR-ABL1), IKZF1, ΔCDKN2A/B, and ΔPAX5 were similarly treated. Dasatinib (20 nM) reduced the proliferation of #2018-136 cells by 56%; ruxolitinib or abemaciclib (0.1 μM) had no effect. The das+rux combination resulted in 63% decrease in proliferation, which is likely due to inhibitory action of dasatinib. IODVA1 (0.5 μM) reduced the proliferation of these cells by 78%. When tested in the colony formation assay, IODVA1 (1 μM) reduced the number of colonies by 60% ($p = 0.001$) (**Fig. S3A,** middle panels**)**.

Original $CD19^+$ cells from patient #2017-129 with Ph^+ (BCR-ABL1; T315I) and mutated SETD2, SF3B1, and TP53 who relapsed after initial treatment were treated with vehicle control, dasatinib, ruxolitinib, (das + rux), and IODVA1. As expected dasatinib, ruxolitinib, or the combination had no effect on proliferation of the $CD19^+$ cells (**Fig. S3A,**

right panel). In contrast, IODVA1 at 1 μM but not at 0.2 μM reduced the CD19$^+$ B-ALL cell counts by 80%. Additionally, we confirmed that IODVA1 does not exert toxic effects to cells of normal stroma (**Fig. S3A**, right panel arrows). Thus, IODVA1 decreases the proliferation of Ph$^+$ B-ALL (BCR-ABL1) primary cells including cells expressing the TKI-resistant T315I mutant consistent with our findings that Ph$^+$ B-ALL (BCR-ABL1) model cells express high levels of VAV3.

The fact that #2017-58 cells did not respond to IODVA1 as well as the other two patient samples is probably due to the low VAV3 expression and level of phosphorylation (**Fig. 6B**) and the existence of other genetic mutations (*e.g.* P2RY8-CRLF2) that promote cell growth independently of VAV3.

Ph-like B-ALL. Our cohort of samples contained numerous cases of Ph-like disease with a diverse series of genetic aberrations. Ph-like ALL is a high-risk subset of leukemia that shares many characteristics of Ph$^+$ B-ALL and contains a variety of genomic alterations that activate kinase and cytokine signaling but where BCR-ABL1 is not expressed (4-6). Our Ph-like patient derived cells generally responded positively to the treatment they received (**Fig. S3B**). At 1 but not 0.2 μM, IODVA1 reduced cell proliferation by at least 95%. The exception was sample #2018-132 which did not respond to any treatment including the CDK4/6 selective inhibitor abemaciclib. IODVA1 (0.5 μM) reduced the proliferation of these cells by 25% like the other FDA approved drugs. Thus, IODVA1 decreases the proliferation of majority of Ph-like B-ALL primary cells despite their heterogenous genetic lesions.

MLL-rearranged B-ALL. Cells from #2018-190, an MLL/AF9 fusion, were also treated with the same drugs. **Fig. S3C** shows that these cells resisted dasatinib, ruxolitinib, and the

combination. IODVA1 (0.5 μM) and abemaciclib (0.1 μM) decreased proliferation of these cells by 50 and 43%, respectively. Similarly, cells from relapsed patient #2016-116 with MLL t(1; 11), t(6; 6) responded very well to IODVA1 either with or without combined SCF/Flt3L/IL-7 cytokine supplementation.

IODVA1 decreases RAC activity and downstream signaling. To determine the effective concentration that decreases RAC activation (7, 8), p190-BCR-ABL1 Ba/F3 cells (9-11) were incubated with IODVA1 (0.1 – 10 μM) for 1 h, followed by GST-PAK-GBD pulldown. **Fig. S4A** shows that IODVA1 decreases levels of active RAC with an IC50 of 1 μM. We also tested the activation of the two related GTPases, CDC42 and RHOA. IODVA1 (3 μM) decreases the levels of active CDC42 by 60% at 15 min and totally inhibits it at 30 min incubation time. Pull-downs with the Rho-binding domain of Rhotekin show that IODVA1 has no effect on RHOA activation (**Fig. S4B)**, consistent with our previous observations (3).

IODVA1 does not interfere with RAC-specific GAP or GDI functions. RAC activity and signaling (12-22) is regulated by RAC-specific GAPs, GDIs, and GEFs. We argued that the decrease in RAC activity might be caused by IODVA1 targeting one RAC regulator. First, we tested *in vitro* if IODVA1 stimulates the activity of the RAC negative regulator p50GAP. RAC was loaded with the fluorescently-labeled tamraGTP and the stimulated increase in GTP-hydrolysis by the C-terminal GAP homology of p50GAP was monitored by stopped-flow fast kinetics for 3 s. **Fig. S4E** shows that the initial decrease in fluorescence, which is coupled to GTP-hydrolysis, does not change in the presence (k_{obs} =

5.7) or absence (k_{obs} = 6.6) of high IODVA1 concentration (50 μM). We thus conclude that IODVA1 does not stimulate the activity of p50GAP to explain the observed decrease in RAC activity.

Second, we tested if IODVA1 increases the detachment of RAC1 from membranes thus making it unavailable for activation by GEFs. We studied the displacement of prenylated RAC1-GDP from synthetic liposomes by GST-RhoGDI1 in the presence and absence of IODVA1 using liposome sedimentation assay (23). GST-RhoGDI1 (4 μM) was added to liposome containing phosphatidylinositol 4,5-bisphosphate (PIP_2) and prenylated RAC1-GDP (1 μM) in the absence or presence of IODVA1 and further incubated on ice for 30 min. Samples were centrifuged at 20,000 x g for 20 min at 4°C and pellet (p) and supernatant (s) fractions were collected and immunoblotted for RAC1. Addition of IODVA1 (2 μM) did not affect prenylated-RAC1 displacement by RhoGDI1 from liposomes (**Fig. S4F,** lanes 2 and 3). We then measured the interaction between fluorescently labeled RAC1 and RhoGDI1 using stopped-flow fast kinetics techniques. The stopped-flow data show that the observed binding affinity between the two proteins did not significantly change in the presence of IODVA1 (k_d = 0.078 μM) or vehicle control (k_d = 0.1 μM), even though the two proteins interact with a different amplitude (**Fig. S4G)**. Taken together, IODVA1 does not interfere with RhoGDI binding to RAC1 or with its ability to extract prenylated-RAC1 from PIP_2-containing membranes.

Vav3-deficient leukemic cells do not respond to IODVA1 in vitro and in vivo. To further validate VAV3 as target of IODVA1, we studied the effects of IODVA1 on leukemic cells from the Vav3-deficient (*Vav3*$^{-/-}$) mice previously published (9, 24, 25). We argued that if

IODVA1 targets VAV3, then *Vav3*$^{-/-}$ cells should be significantly less sensitive to its action. We tested if the lack of response to IODVA1 by *Vav3*$^{-/-}$ cells holds *in vivo*. We transplanted wild-type (*Vav3*$^{+/+}$) or *Vav3*$^{-/-}$ LDBM cells transduced with p190-BCR-ABL1 retrovirus into lethally irradiated C57BL/10 mice (N = 5 per group), waited 21 days for the leukemia to develop, and treated the mice with either vehicle control or IODVA1 administered through osmotic pumps as before (**Fig. S5A)**. Because the mice died shortly post leukemia transplantation, all groups were treated for 14 days only, *i.e.* all treatments started at day 21 and ended by day 35. Kaplan-Meier survival plots show that, as expected, mice transplanted with wild-type leukemia and treated with vehicle control die between days 33 and 38. Mice treated with IODVA1 survive until day 60, *i.e.* 25 days after treatment has ended. Compared to mice receiving vehicle control where leukemic progenitors constituted 23.8% of peripheral blood cells, mice treated with IODVA1 showed drastic reduction of levels of leukemic progenitors to 5% and 2% after one week and two weeks of IODVA1 treatment, respectively (**Fig. S5B,** dark lilac bars**)**. These in vivo data are consistent with **Fig. S1E** and with the hypothesis that IODVA1 eliminates leukemic progenitor cells responsible for disease propagation despite the short treatment time. Mice transplanted with *Vav3*$^{-/-}$ leukemia and treated with vehicle control or IODVA1 (**Fig. S5A**, grey and light lilac lines, respectively) die between days 34 and 42 and days 40 and 44, respectively. Thus, IODVA1 had no significant effect on *Vav3*$^{-/-}$ leukemic mice ($p = 0.41$). *Vav3*$^{-/-}$ leukemic mice have an increased survival compared to their wild-type leukemic counterpart, consistent with our previous observations (24). This can also be seen in the level of peripheral blood leukemic progenitor cells that kept increasing in *Vav3*$^{-/-}$ leukemic mice treated with IODVA1 or vehicle control (**Fig. S5B**).

The observation that mice engrafted with *Vav3*$^{-/-}$ leukemia die by day 44 while mice engrafted with normal leukemia and treated with IODVA1 survive until day 60 even after treatment has ended suggests that leukemic cells in the *Vav3*$^{-/-}$ background rely on alternative pathways to survive. To test this hypothesis, we studied by phospho-flow cytometry the activity of several effectors in BM cells from one-week treated mice. The phosphorylation levels of the VAV3/RAC effectors JNK and PAK1 are severely reduced in IODVA1-treated mice regardless of VAV3 status (**Fig. S5C**). The levels of pJNK and pPAK1 in *Vav3*$^{-/-}$ leukemia are not affected by IODVA1 and are similar to IODVA1-treated wild-type leukemia. Interestingly, the phosphorylation levels of the non-VAV3/RAC effectors AKT and STAT3 are not only unaffected by IODVA1 in wild-type leukemia as seen before in **Fig. 3B & 6A** but significantly increased in *Vav3*$^{-/-}$ leukemia. This observation suggests that *in vivo*, *Vav3*$^{-/-}$ leukemic cells are not only unresponsive to IODVA1 but rely among others on STAT3 signaling for survival.

ACKNOWLEDGMENTS. We thank Drs. Marie-Dominique Filippi, Damien Reynaud, Daniel Starczynowski, and their lab members for valuable advice, Dr. Yuting Tang for help with figure preparation, and Ashley Wellendorf for retrovirus preparation and mouse colony maintenance. We thank Drs. Xosé R. Bustelo and Myriam Cuadrado (University of Salamanca, Spain) for sharing VAV3 antibody and VAV3 expression constructs. This project was funded by NIH grants (R01CA115611 to N.N.N. and R01CA237016 to N.N.N. and J.A.C., and R50CA211404 to M.W.), an American Society of Hematology Bridge Award (N.N.N.), Translational Research Program grants from the Leukemia Lymphoma Society (6076-14 and 6264-13 to N.N.N. and J.A.C.), an Affinity Group Award from the Cincinnati Cancer Center (N.N.N. and J.A.C), a FY19 Cincinnati Children's Innovation Fund award (N.N.N and J.A.C.), a CancerFree Kids Pediatric Cancer Research Alliance award (N.N.N.), an award from the National Center for Advancing Translational Sciences of the National Institutes of Health, under Award Number 2UL1TR001425-05A1 (N.N.N.), the German Research Foundation (Deutsche Forschungsgemeinschaft; AH 92/8-1 to M.A. and M.R.A.), the European Network on Noonan Syndrome and Related Disorders (NSEuroNet, 01GM1621B to O.H.F.K. and M.R.A.), the German Federal Ministry of Education and Research (BMBF) – German Network of RASopathy Research (GeNeRARe, 01GM1902C to M.R.A.), and the International Research Training Group 1902 Intra- and Interorgan Communication of the Cardiovascular System (IRTG 1902-P6 to M.B. and M.R.A.).

Supplemental Methods

Plasmids, Cell Lines, and Reagents: Plasmid set for purification of fixed-arm carrier fusions pMalX (A-E) was a kind gift from Dr. Lars C. Pedersen (NIEHS), pET28b-N_9-MBP-mOrange plasmid (#29748) was from Addgene (Watertown, MA), chaperone co-expression plasmid set was from TaKaRa Bio USA (Mountain View, CA). Primers were from Integrated DNA Technologies (Coralville, IA). Restriction enzymes, polymerases, cloning assembly kits and competent cells were from New England Biolabs (Ipswitch, MA). Cytokines were from Peprotech (Rocky Hill, NJ).

Cell lines were purchased from American Type Culture Collection (ATCC, Manassas, VA) or German Collection of Microorganisms and Cell Cultures (DSMZ, Braunschweig, Germany) and were not further authenticated. The cell lines are not registered as commonly misidentified cell lines. Cells are routinely checked for mycoplasma by PCR. Ba/F3 cells were cultured in RPMI (ThermoFisher) supplemented with 10% FBS and IL-3 (10 ng/ml), NALM-1 cells were maintained in RPMI supplemented with 15% FBS. HEK293T cells were maintained with DMEM supplemented with 10% FBS and 1% penicillin/streptomycin. All cell lines were cultured at 37°C in a 5% CO_2 humidified incubator. Cell viability was assessed by trypan blue exclusion as previously described (3).

The following antibodies were used: GAPDH (#627408, GeneTex, Irvine, CA), pERK1/2 (#4370), pAKT (#9271 and #9018), c-Abl (#2862), CDC42 (#2462), RHOA (#2117), pPAK1/2 (#2601S), pS6 (#4851S), PAK1 (#2602S), pBAD (#4366), and BAD (#9292), pCrkL (#3181), CrkL (#3182), anti-mouse HRP (#7076), anti-rabbit HRP (#7074) were from Cell Signaling Technologies (Danvers, MA), pVAV1 (Y174) (#ab76225),

pVAV3(Y173) (#ab109544), total VAV3 (#ab203315) were from Abcam (Cambridge, MA). Total VAV3 antibody was also graciously shared by Dr. Xosé Bustelo's laboratory, pJNK (Alexa Fluor 647 conjugated, #562481), p-p38 (PE-conjugated, #612565), RAC2 (#610850), pSTAT3 (#55385), and pSTAT5 (Alexa Fluor 647 conjugated, #612599), and B220 APC-Cy7 antibody (#552094) were from BD Bioscience (San Diego, CA), anti-phosphotyrosine antibody (#05321) was from Millipore Sigma (St. Louis, MO), p4EBP1 (PE-conjugated, #12-9107-42) was from ThermoFisher. Anti-human CD19 APC-Cy7 (#363009) and anti-human CD45 FITC (#304005) were from BioLegend (San Diego, CA). Lipids (Phosphatidylserine (PS), Phosphatidylcholine (PC), phosphatidylethanolamine (PE) and sphingomyelin (SM), and phosphatidylinositol 4,5-bisphosphate (PIP_2) for membrane displacement assays were from Avanti Polar Lipids (Darmstadt, Germany).

Chemicals: IODVA1 and its biotinylated analog were synthesized by WuXi AppTech (Hong Kong) from 2-guanidinobenzimidazole and purified as described (3) but at 20 °C. Imatinib methanesulfonate salt (#I-5508), dasatinib (#D-3307), and ponatinib (#P-7022) were from LC Laboratories (Woburn, MA), ruxolitinib (#S1378) from Selleck Chemicals (Houston, TX).

Osmotic pump implantation and survival analysis: Osmotic pumps (ALZET) were prepared according to manufacturer's protocol. When leukemic burden reached 12-14% in peripheral blood, mice were stratified into different groups. Leukemic mice were anesthetized and osmotic pumps with indicated drugs were surgically implanted subcutaneously on the back of the mouse. Each pump lasted 14 days.

Impaired mobility/limb paralysis, interference with vital physiological functions and labored breathing, hunched abnormal posture, significant weight loss and hematological signs indicative of organ failure were used as humane endpoints.

Viral Particle Production, Transduction and Transplantation: Production of lentivirus and retrovirus for stable transduction of murine and human cells were done as described previously (26). Retroviral and lentiviral vectors, viral transduction of cell lines and mouse LDBM, and transplantation of transduced leukemic cells were previously described (24). For VAV3 rescue experiments, low density bone marrow cells from wild-type (*Vav3*$^{+/+}$) or *Vav3*$^{-/-}$ mice were transduced with bicistronic retroviral vector encoding p190-BCR-ABL1-IRES-YFP and sorted for YFP^{+} 48 h post-transduction. Cells were then transduced with lentiviral particles encoding either empty vector, full-length WT or mutant VAV3 (pCDH1-MCS1-EF1-copGFP). Cells were sorted for GFP^{+}/YFP^{+} and treated with IODVA1 at the indicated concentrations. Cell cycle was analyzed at 18 h post BrdU incorporation.

Flow Cytometry and Cell Cycle Analysis: Red blood cells were removed from the peripheral blood samples using fixative-free lysis buffer (BD Biosciences). After a single wash in PBS, cells were stained with anti-B220 APC-Cy7 antibody. Stained cells were washed once and analyzed by flow cytometry. Cell cycle was analyzed via *in vitro* BrdU incorporation (BD). Briefly, leukemic progenitors were incubated with 1 mM BrdU solution for 45 minutes, cells were further fixed and permeabilized. DNAse treatment was done according to the instructions and stained with anti BrdU and apoptosis was analyzed

by 7-AAD staining through flow cytometry analysis. Analysis was performed using FACSCanto (BD Biosciences) at the CCHMC Research Flow Cytometry Core.

SDS-PAGE, Pull-down Assays, and Immunoblotting: For analysis of GTPase status, exponentially growing cells, treated with either vehicle or IODVA1 at the indicated concentrations and time points, were subjected to active GTPase pulldown kits using GST-PAK1-GBD or GST-Rhotekin (ThermoFisher). Protein complexes were separated on SDS-PAGE and immunoblotted with anti-RAC1, anti-CDC42 and anti-RHOA antibodies that came with the kit. For analysis of VAV3 binding to biotinylated IODVA1, recombinant VAV3 or lysates from PDX specimens were subjected to neutravidin pulldowns, followed by SDS-PAGE and immunoblotting analyses.

For analysis of expression and cell signaling, cells were subjected to lysis and immunoblotting, as described previously (24, 26). Relative signals were normalized to the unstimulated conditions after normalization to the total protein amount. Quantification was performed using Li-COR Image Studio (Lincoln, NE) or ImageJ (NIH, Bethesda, MD).

Recombinant Protein Cloning, Expression and Purification: For bacterial expression, full-length VAV3 was cloned as an MBP-fusion protein into pMalX(E) vector with N-terminal AAAA, AAAASEF or AAAASEFGS linkers using HiFi assembly (NEB). N-VAV3 (aa 1-575) was cloned as a His_6-tagged protein into pProEx-HTB vector. All constructs were verified using Sanger sequencing using CCHCM DNA Core. To minimize aggregation and improve on quality of purified protein, the expression clones were tested with chaperone plasmids according to the manufacturer's protocol (Takara Bio USA).

For production of recombinant full-length and truncated VAV3, plasmids were co-transformed with chaperone plasmid Gro7 groEL-groES in BL21 (DE3) or T7 Express. Cultures were grown in LB, supplemented with metal mix. Protein was purified using Ni-IMAC chromatography, followed by size-exclusion gel filtration (HiLoad Superdex 200 16/60, GE Healthcare, Chicago, IL). Post SDS-PAGE analysis, fractions containing VAV3 were pooled, concentrated to ~10 mg/mL and flash frozen in liquid nitrogen. Final yield was 10-20 mg per 6 L of culture. Recombinant LARG (DH/PH) was purified as His_6-MBP-fusion protein using IMAC, followed by size-exclusion gel filtration as above.

Recombinant Protein Cloning, Expression and Purification: Human *RAC1* (GenBank accession n° NM_006908.4) was subcloned as N-terminally His_6-tagged construct into pFastBacHTB vector (ThermoFisher). Full-length human RAC1 was purified from baculovirus. RAC1 was produced in TNAO38 insect cells and purified using Ni-IMAC chromatography.

RhoGDI Extracting Prenylated RAC1 from Liposomes: Displacement of prenylated-RAC1-GDP from synthetic liposomes by GST-RhoGDI1 in the presence and absence of IODVA1 was studied using liposome sedimentation assay as in (23). Briefly, liposomes were generated by using a defined composition of lipids (194 μg) containing 39% w/w phosphatidylethanolamine, 16% w/w phosphatidylcholine, 36% w/w phosphatidylserine, 4% sphingomyelin, and 5 % w/w phosphatidylinositol 4,5-bisphosphate. Prenylated RAC1-GDP (1 μM) was added to liposomes suspended in protein buffer (20 mM Hepes, pH 7.4, 150 mM NaCl, 5 mM $MgCl_2$, 3 mM DTT) and incubated for 20 min on ice. GST-

RHOGDI1 (2 μM) in the absence or presence of IODVA1 was added to the liposome/prenylated RAC1 and further incubated on ice for 30 min. The samples were then centrifuged at 20,000 x g for 20 min at 4 °C. Pellet and supernatant fractions were collected, separated on SDS-PAGE and immunoblotted for RAC1.

Stopped-flow Spectrometry: GTPase assay and nucleotide exchange reaction were performed with a SF-61 stopped-flow instrument (TgK Scientific, Bradford-on-Avon, United Kingdom) as described (27) The excitation wavelengths were 543 nm and 362 nm for tamraGTP and mantGppNHp, respectively. For GTPase assay, equal volumes (600 μl) of 0.2 μM RAC1- tamraGTP and 10 μM of p50GAP were used. GTPase assay as well the protein-protein interaction were performed in presence of 5% DMSO.

Microscale Thermophoresis (MST): Purified VAV3, LARG or RAC (1 μM) were incubated with the indicated concentrations of IODVA1 at room temperature for 30 min. We argued that if IODVA1 binds other targets, it is likely to bind to proteins with common motives and we tested the DH/PH domain of LARG. Samples were loaded into Zero Background MST Premium Coated capillaries and binding events were measured on Monolith NT.LabelFree (NanoTemper Technologies, Munich, Germany). Binding data were analyzed using Thermophoresis or Thermophoresis with Temperature Jump analysis. Data were normalized using fraction-bound binding. The 95% confidence interval for K_d values was 0.27 to 0.98 μM for VAV3, 5.9 to 10.37 μM for LARG, and 19.6 to 105.8 μM for RAC.

CFU-proB Assay: B-cell lineage colony-forming units (CFU-proB) were quantified post 9-day culture of leukemic BM cells or sorted for p190-BCR-ABL1–expressing B-cell progenitors in M3134 methylcellulose (StemCell Technologies, Cambridge, MA) supplemented with 30% FBS, 2 mM L-glutamine and 1% penicillin-streptomycin (Invitrogen), 100 μM β-mercaptoethanol (Fisher-Scientific), 1% BSA (Sigma-Aldrich), 20 ng/mL of recombinant mIL-7 and 100 ng/mL of recombinant mSCF (PeproTech).

Ex vivo Drug Treatment of Clinical Samples: For analysis of proliferation during drug treatment, spleen preparations from mice successfully engrafted with B-ALL were co-cultured with MS-5 or OP9 stroma in MEMα media supplemented with 20% FBS and 10 ng/mL recombinant human SCF (Kit-L), Flt3L, and IL-7 (KF7). Drug treatment was started 24 h after initial seeding. Co-cultures were collected by trypsinization after 7 days and cell counts were performed with trypan blue. Flow Cytometry was performed with anti-mouse CD45-APC-Cy7 (#557659), anti-human CD45-FITC (#561865) (BD Biosciences, Franklin Lakes, NJ), anti-human CD19-VioBlue (#130-113-172) and 7-AAD (#130-111-568) (Miltenyi Biotec, Bergisch Gladbach, Germany) to determine percentage of human ALL in the cultures. Total absolute ALL cell numbers were determined by multiplying cell counts by percentage human ALL cells.

Statistical Analysis: Statistical analyses were performed in Prism v.8 (GraphPad Software, San Diego, CA). Additionally, *Essential Statistics for the Pharmaceutical Sciences* (Philip Rowe) was consulted to choose appropriate statistical tests. Comparison of two groups was carried out using Student's t-test, comparison of datasets with more than

two groups was carried out using One- or Two-way ANOVA, as appropriate, with recommended multiple comparisons tests. Animal studies were planned to provide 60-80% power for a target effect size of 1.2-1.5. A sample size of 5-6 mice per group and experimental replicate was calculated and used; see figure legends for specific sample sizes, noted as "N." Animals with higher number of circulating leukemic progenitors were assigned to the treatment group, animals with lower numbers of progenitors were assigned to the vehicle control group. No blinding or randomization was done. For cell viability and proliferation studies in liquid culture and methylcellulose/soft agar of p190-BCR-ABL1-transformed cells, experiments were performed in triplicates (3 technical replicates) at least 3 times (3 independent experiments). Pull-down, immunoprecipitation and immunoblot experiments were repeated at least twice. In figures summarizing animal survival data, each point on a Kaplan-Meier curve represents one mouse, in figures summarizing cell experiments, data are presented as mean ± standard deviation, unless otherwise noted in the legend. No data were excluded from analysis. Alpha was set to 0.05. Two-sided tests were used. Results of statistical tests are presented as p-value nominations, where * corresponds to $p \leq 0.05$, ** to $p \leq 0.01$, *** to $p \leq 0.001$, **** to $p < 0.0001$.

Supplemental References

1. Warmuth M, Kim S, Gu XJ, Xia G, Adrian F. Ba/F3 cells and their use in kinase drug discovery. Curr Opin Oncol. 2007;19(1):55-60.

2. Sonta SI, Minowada J, Tsubota T, Sandberg AA. Cytogenetic study of a new Ph1-positive cell line (NALM-1). Journal of the National Cancer Institute. 1977;59(3):833-7.

3. Gasilina A, Premnauth G, Gurjar P, Biesiada J, Hegde S, Milewski D, et al. IODVA1, a guanidinobenzimidazole derivative, targets Rac activity and Ras-driven cancer models. PLoS One. 2020;15(3):e0229801.

4. Tasian SK, Loh ML, Hunger SP. Philadelphia chromosome-like acute lymphoblastic leukemia. Blood. 2017;130(19):2064-72.

5. Roberts KG, Mullighan CG. Genomics in acute lymphoblastic leukaemia: insights and treatment implications. Nat Rev Clin Oncol. 2015;12(6):344-57.

6. Pui CH, Roberts KG, Yang JJ, Mullighan CG. Philadelphia Chromosome-like Acute Lymphoblastic Leukemia. Clin Lymphoma Myeloma Leuk. 2017;17(8):464-70.

7. Nieborowska-Skorska M, Kopinski PK, Ray R, Hoser G, Ngaba D, Flis S, et al. Rac2-MRC-cIII-generated ROS cause genomic instability in chronic myeloid leukemia stem cells and primitive progenitors. Blood. 2012;119(18):4253-63.

8. Wei J, Wunderlich M, Fox C, Alvarez S, Cigudosa JC, Wilhelm JS, et al. Microenvironment determines lineage fate in a human model of MLL-AF9 leukemia. Cancer Cell. 2008;13(6):483-95.

9. Thomas EK, Cancelas JA, Zheng Y, Williams DA. Rac GTPases as key regulators of p210-BCR-ABL-dependent leukemogenesis. Leukemia. 2008;22(5):898-904.

10. Harnois T, Constantin B, Rioux A, Grenioux E, Kitzis A, Bourmeyster N. Differential interaction and activation of Rho family GTPases by p210bcr-abl and p190bcr-abl. Oncogene. 2003;22(41):6445-54.

11. Sahay S, Pannucci NL, Mahon GM, Rodriguez PL, Megjugorac NJ, Kostenko EV, et al. The RhoGEF domain of p210 Bcr-Abl activates RhoA and is required for transformation. Oncogene. 2008;27(14):2064-71.

12. Jaffe AB, Hall A. Rho GTPases: biochemistry and biology. Annu Rev Cell Dev Biol. 2005;21:247-69.

13. Loirand G, Pacaud P. The role of Rho protein signaling in hypertension. Nat Rev Cardiol. 2010;7(11):637-47.

14. Newey SE, Velamoor V, Govek EE, Van Aelst L. Rho GTPases, dendritic structure, and mental retardation. J Neurobiol. 2005;64(1):58-74.

15. Vigil D, Cherfils J, Rossman KL, Der CJ. Ras superfamily GEFs and GAPs: validated and tractable targets for cancer therapy? Nat Rev Cancer. 2010;10(12):842-57.

16. Zandvakili I, Lin Y, Morris JC, Zheng Y. Rho GTPases: Anti- or pro-neoplastic targets? Oncogene. 2017;36(23):3213-22.

17. Coleman ML, Marshall CJ, Olson MF. RAS and RHO GTPases in G1-phase cell-cycle regulation. Nat Rev Mol Cell Biol. 2004;5(5):355-66.

18. Kiosses WB, Shattil SJ, Pampori N, Schwartz MA. Rac recruits high-affinity integrin alphavbeta3 to lamellipodia in endothelial cell migration. Nat Cell Biol. 2001;3(3):316-20.

19. Sundaresan M, Yu ZX, Ferrans VJ, Sulciner DJ, Gutkind JS, Irani K, et al. Regulation of reactive-oxygen-species generation in fibroblasts by Rac1. Biochem J. 1996;318 (Pt 2):379-82.

20. Bustelo XR. RHO GTPases in cancer: known facts, open questions, and therapeutic challenges. Biochem Soc Trans. 2018;46(3):741-60.

21. Ridley AJ. Rho GTPase signalling in cell migration. Curr Opin Cell Biol. 2015;36:103-12.

22. Steffen A, Ladwein M, Dimchev GA, Hein A, Schwenkmezger L, Arens S, et al. Rac function is crucial for cell migration but is not required for spreading and focal adhesion formation. J Cell Sci. 2013;126(Pt 20):4572-88.

23. Zhang SC, Gremer L, Heise H, Janning P, Shymanets A, Cirstea IC, et al. Liposome reconstitution and modulation of recombinant prenylated human Rac1 by GEFs, GDI1 and Pak1. PLoS One. 2014;9(7):e102425.

24. Chang KH, Sanchez-Aguilera A, Shen S, Sengupta A, Madhu MN, Ficker AM, et al. Vav3 collaborates with p190-BCR-ABL in lymphoid progenitor leukemogenesis, proliferation, and survival. Blood. 2012;120(4):800-11.

25. Thomas EK, Cancelas JA, Chae HD, Cox AD, Keller PJ, Perrotti D, et al. Rac guanosine triphosphatases represent integrating molecular therapeutic targets for BCR-ABL-induced myeloproliferative disease. Cancer Cell. 2007;12(5):467-78.

26. Lee LH, Gasilina A, Roychoudhury J, Clark J, McCormack FX, Pressey J, et al. Real-time genomic profiling of histiocytoses identifies early-kinase domain BRAF alterations while improving treatment outcomes. JCI Insight. 2017;2(3):e89473.

27. Nouri K, Fansa EK, Amin E, Dvorsky R, Gremer L, Willbold D, et al. IQGAP1 Interaction with RHO Family Proteins Revisited: KINETIC AND EQUILIBRIUM EVIDENCE FOR MULTIPLE DISTINCT BINDING SITES. J Biol Chem. 2016;291(51):26364-76.

Figure S1: IODVA1 inhibits the proliferation and survival of BCR-ABL1 expressing cells *in vitro* and *in vivo* and eradicates leukemia-propagating cells in secondary transplants. (**A**) Ba/F3 cells transduced with p190-BCR-ABL1 (lilac squares), p210-BCR-ABL1 (light lilac triangles), or empty vector (black circles) were grown in the presence of vehicle control or IODVA1 at 1 and 3 μM and counted daily for 3 days using trypan blue exclusion. Mean ± SD of a representative experiment done in triplicates is shown. (**B**) IODVA1-dependent survival curves of empty vector (black circles), p190-BCR-ABL1 expressing Ba/F3 cells grown in the absence (orange circles) or presence (lilac circles) of IL-3 and of Nalm-1 cells. Fitting of the data was done in Prism version 8.4. Combined mean ± SEM of all experiments is shown. (**C**) p190-BCR-ABL1 expressing Ba/F3 cells were allowed to grow for 1 day, treated with IODVA1 (IO1, 1 μM) for 1 day, and washed. Cells were counted for 7 days using trypan blue exclusion. Mean ± SD of a representative experiment done in triplicates is shown. (**D**) Ba/F3 cells stably expressing p190-BCR-ABL1 were subjected to colony formation assay in soft agar in the presence of DMSO or IODVA1 (1 or 10 μM). Colonies were allowed to form for 10 days then stained with iodonitrotetrazolium. Data shown are representative of three independent experiments done in triplicates. Note the smaller colony size in 1 μM IODVA1 treatment group. (**E**) Leukemic burden (%) of treated mice before treatment and at the indicated treatment time was analyzed by flow cytometry of bone marrow aspirates as population containing $B220^+/CD43^+$ pro-B cells. N = 3 mice per group (**F**) Count (%) of residual leukemic ($EGFP^+$-BCR-ABL1) cells in peripheral blood at weeks 3 and 5 for the secondary transplant mice from **Fig. 1D**. (**G**) Kaplan-Meier survival plot of secondary mice transplants with the 0.3 x 10^6 cell-dilution. (**H**) Count (%) of residual leukemic ($EGFP^+$-

BCR-ABL1) cells in peripheral blood at weeks 3 and 5 for the secondary transplant mice from **(G)**. (**I & J**) like **G & H** but with the 0.1 x 10^6 cell-dilution. N = 5 mice per group in F-J. * $p \leq 0.05$ using One-way ANOVA with Tukey's multiple comparison test.

Figure S2: IODVA1 does not affect p190-BCR-ABL1 phosphorylation status. Ba/F3 cells expressing p190-BCR-ABL1 were treated with vehicle control, imatinib (IM, 1 μM), or IODVA1 (3 μM) for 4 h. Lysates were immunoprecipitated with ABL1 antibody and the protein complex separated on SDS-PAGE and immunoblotted for phosphotyrosine (pY) and BCR-ABL1 (c-Abl) and quantified. Lysates were also immunoblotted for pCrkl and total Crkl and quantified. IP – immunoprecipitation, IB – immunoblotting.

Figure S3: IODVA1 reduces survival of leukemic cells derived from relapsed and *de novo* Ph$^+$, Ph-like and MLL pediatric patients. Patient derived xenograft (PDX) cells were co-cultured *ex vivo* on MS-5 or OP-9 stromal cells and treated with dasatinib (Das, ABL1-inhibitor), ruxolitinib (Rux, JAK-inhibitor), combination of dasatinib and ruxolitinib (Das + Rux), abemaciclib (CDK inhibitor), or IODVA1 at the indicated concentrations and assessed for survival using flow cytometry. (**A**) Representative survival of leukemic cells from patients with Ph$^+$ rearrangements. Cells from sample #2018-136 were also subjected to clonogenic assay. Also note example image from #2017-129 cells treated with control, dasatinib or IODVA1 showing no effect on cells of normal stroma (black arrows) (**B**) Leukemic cells from patients with *de novo* Ph-like leukemia. **(C)** Leukemic cells from MLL/AF9 and relapsed MLL/AF1q patients. (**D**) Cells from PDX Ph-like cells 2016-79 and 2018-132 with the same (IGH-CRLF2; JAK2) rearrangement were

incubated with IODVA1 (1 μM) for 4 h, lysed, and immunoblotted for pVAV3 and VAV3; GAPDH was used as loading control.

Figure S4: IODVA1 decreases RAC signaling but does not interfere with the action of the RAC negative regulators GAP or RhoGDI. (**A**) Levels of active RAC were assessed using PAK-GBD pull-down assay as was done for **Fig. 3A**, but cells were incubated for a fixed amount of time (1 h) at the indicated IODVA1 concentrations. Densitometric quantification of active RAC (RAC•GTP) levels were done using ImageJ (**B**) p190-BCR-ABL1 Ba/F3 cells were treated with IODVA1 (3 μM) and levels of active CDC42 (Cdc42•GTP) and RHOA (Rho•GTP) were assessed by pull-down at the indicated times using GST-PAK-GBD and GST-Rhotekin respectively, followed by immunoblotting. Levels of active GTPase were assessed using ImageJ as in **S3A**. (**C**) p190-BCR-ABL1 Ba/F3 cells were treated with IODVA1 (3 μM) and lysed at the indicated times. Cell lysates were separated on SDS-PAGE and immunoblotted for pPAK1/2 (T423/T402), pBAD (S136), and BAD. GAPDH was used as loading control. **A – C** representative blots of two independent experiments. (**D**) Morphology of GFP^+ leukemic colonies (left panel). Western blot analysis of RAC1 and RAC2 protein expression in $Rac1^{\Delta/\Delta}$+$Rac2^{-/-}$ cells post poly-I:C injections (right panel). (**E**) Intrinsic (blue line) and p50GAP-stimulated GTP-hydrolysis reaction in the presence (red line) or absence (black line) of IODVA1. (**F**) Sedimentation assay of liposomal RAC1-GDP in the presence of RhoGDI1 (4 μM) and IODVA1 (2 μM). RAC1 was visualized by immunoblotting from pellet (p) and soluble (s) fractions. (**G**) Stopped-flow measurement of GDI (10 μM)

interaction with fluorescently labelled RAC1 in the absence (black line) or presence (red line) of IODVA1.

Figure S5: VAV3-deficient leukemia is not responsive to IODVA1. (A) Kaplan-Meier plot showing survival of wild-type or *Vav3*$^{-/-}$ p190-BCR-ABL1 leukemic mice post-treatment with osmotic pumps implanted subcutaneously and carrying vehicle control or IODVA1 (IO1, 1 mM). N = 5 mice per treatment group. (**B**) Count (% leukemic progenitors in peripheral blood) of residual leukemic (EGFP^{+}-BCR-ABL1) cells at week 1 and 2 post-treatment for mice from **(A)**. (**C**) Bar graph of pharmacodynamic assessment of leukemic progenitor cells (%) from wild-type or *Vav3*$^{-/-}$ mice with p190-BCR-ABL1 leukemia and treated with vehicle control (black and light grey) or IODVA1 (IO1, dark and light lilac) following 2-week treatment using phospho-flow analysis of the indicated effectors. (**D**) Bar graph summarizing results of the phospho-flow cytometric analysis of the indicated signaling molecules in the leukemic progenitor bone marrow aspirates of wild-type or *Vav3*$^{-/-}$ mice treated with IODVA1. (**E**) IODVA1-dependent survival curves of empty vector- (black lines), wild type full length VAV3- (light lilac lines) or ΔCH-mutant of VAV3- (lilac lines) expressing Ba/F3 cells in the empty vector- or p190-BCR-ABL1-transduced background. (**F**) Results of biotinylated-IODVA1 pull-down from PDX Ph^{+} B-ALL 2018-136 and p190-BCR-ABL1-Ba/F3 cell lysates. Neutravidin bead-bound protein complexes were washed, separated on SDS-PAGE, and immunoblotted for PREX1, VAV3, and VAV1. Results are representative of at least two independent experiments. (**G**) pVAV3 levels in the bone marrow aspirates of PDX 2018-136 engrafted mice treated with vehicle control or dasatinib at the time of sacrifice or IODVA1 (IO1) at the end of

treatment. Representative blot of two experiments, n = 3 mice per group. * $p \leq 0.05$; ** $p \leq 0.01$, *** $p \leq 0.001$, **** $p < 0.0001$ using One-way ANOVA with Tukey's multiple comparison test.

Supplemental Table Legend

Table S1: List of ALL patients with samples available through the Pediatric Leukemia Avatar Program at CCHMC, including those with cytokine receptor, tyrosine kinase (TK), or RAS pathway mutations. Clinical history of TKI treatment is indicated with the TKI received. All samples have confirmed patient-derived xenografts (PDXs) with disease latency as noted. Highlighted samples indicate those selected for Ph+/Ph-like cohort. Patient samples that have established in vitro culture are marked (*). Next-Gen Sequencing (NGS) was performed using the FoundationOne Heme Panel (Cambridge, MA). Other abbreviations: relapsed or refractory (R/R), busulfan (BU), Philadelphia chromosome (Ph^{+}), Philadelphia-like (Ph-like), minimal residual disease (MRD), bone marrow transplant (BMT).

Figure S1

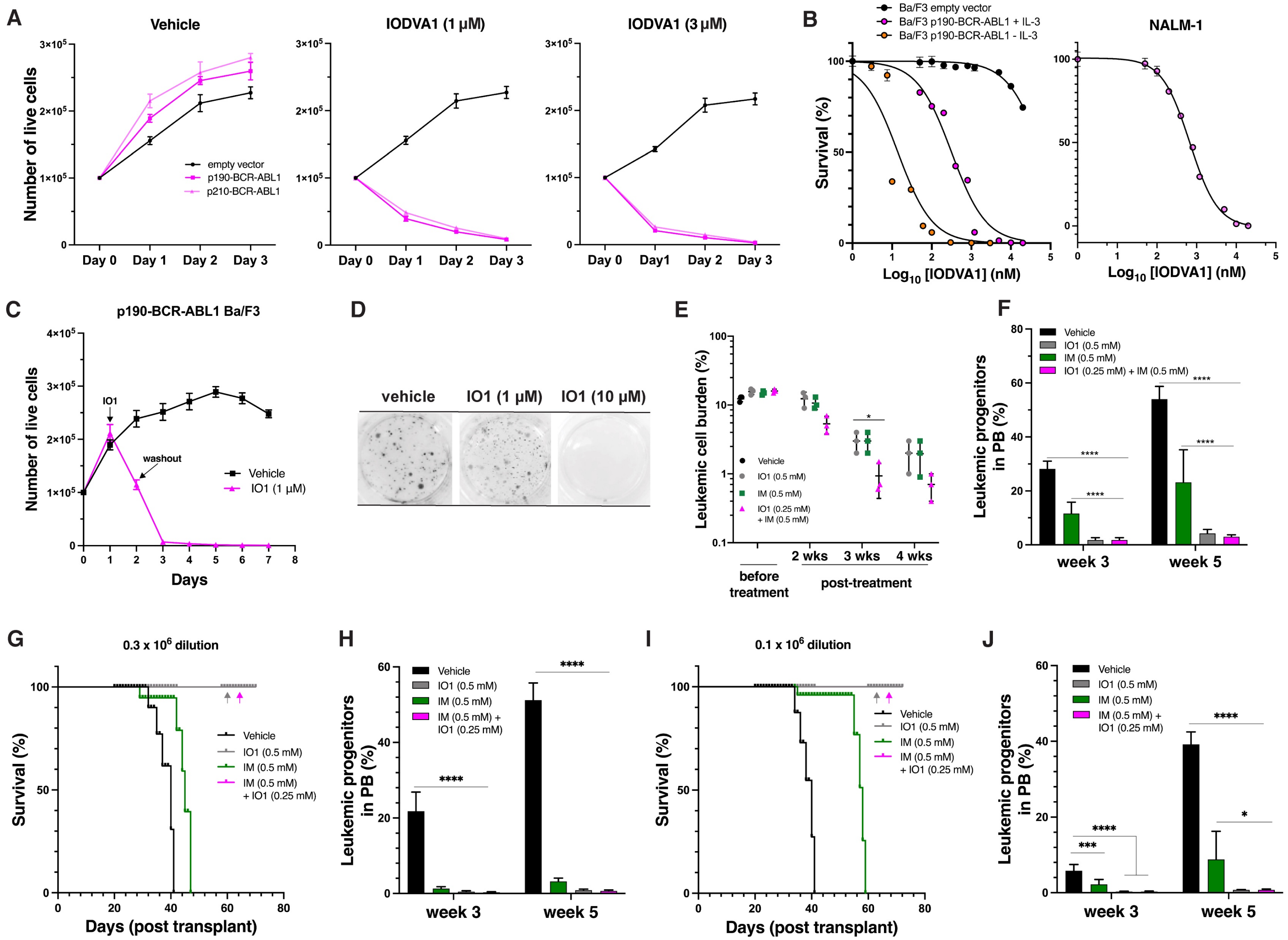
A
Vehicle
IODVA1 (1 μM)
IODVA1 (3 μM)
Number of live cells
empty vector
p190-BCR-ABL1
p210-BCR-ABL1
Day 0
Day 1
Day 2
Day 3
Day1
B
Ba/F3 empty vector
Ba/F3 p190-BCR-ABL1 + IL-3
Ba/F3 p190-BCR-ABL1 - IL-3
NALM-1
Survival (%)
Log10 [IODVA1] (nM)
C
p190-BCR-ABL1 Ba/F3
IO1
washout
Vehicle
IO1 (1 μM)
Days
D
vehicle
IO1 (1 μM)
IO1 (10 μM)
E
Leukemic cell burden (%)
Vehicle
IO1 (0.5 mM)
IM (0.5 mM)
IO1 (0.25 mM) + IM (0.5 mM)
before treatment
2 wks
3 wks
4 wks
post-treatment
F
Vehicle
IO1 (0.5 mM)
IM (0.5 mM)
IO1 (0.25 mM) + IM (0.5 mM)
Leukemic progenitors in PB (%)
week 3
week 5
G
0.3 x 10^6 dilution
Survival (%)
Vehicle
IO1 (0.5 mM)
IM (0.5 mM)
IM (0.5 mM) + IO1 (0.25 mM)
Days (post transplant)
H
Vehicle
IO1 (0.5 mM)
IM (0.5 mM)
IM (0.5 mM) + IO1 (0.25 mM)
Leukemic progenitors in PB (%)
week 3
week 5
I
0.1 x 10^6 dilution
Survival (%)
Vehicle
IO1 (0.5 mM)
IM (0.5 mM)
IM (0.5 mM) + IO1 (0.25 mM)
Days (post transplant)
J
Vehicle
IO1 (0.5 mM)
IM (0.5 mM)
IM (0.5 mM) + IO1 (0.25 mM)
Leukemic progenitors in PB (%)
week 3
week 5

Figure S2

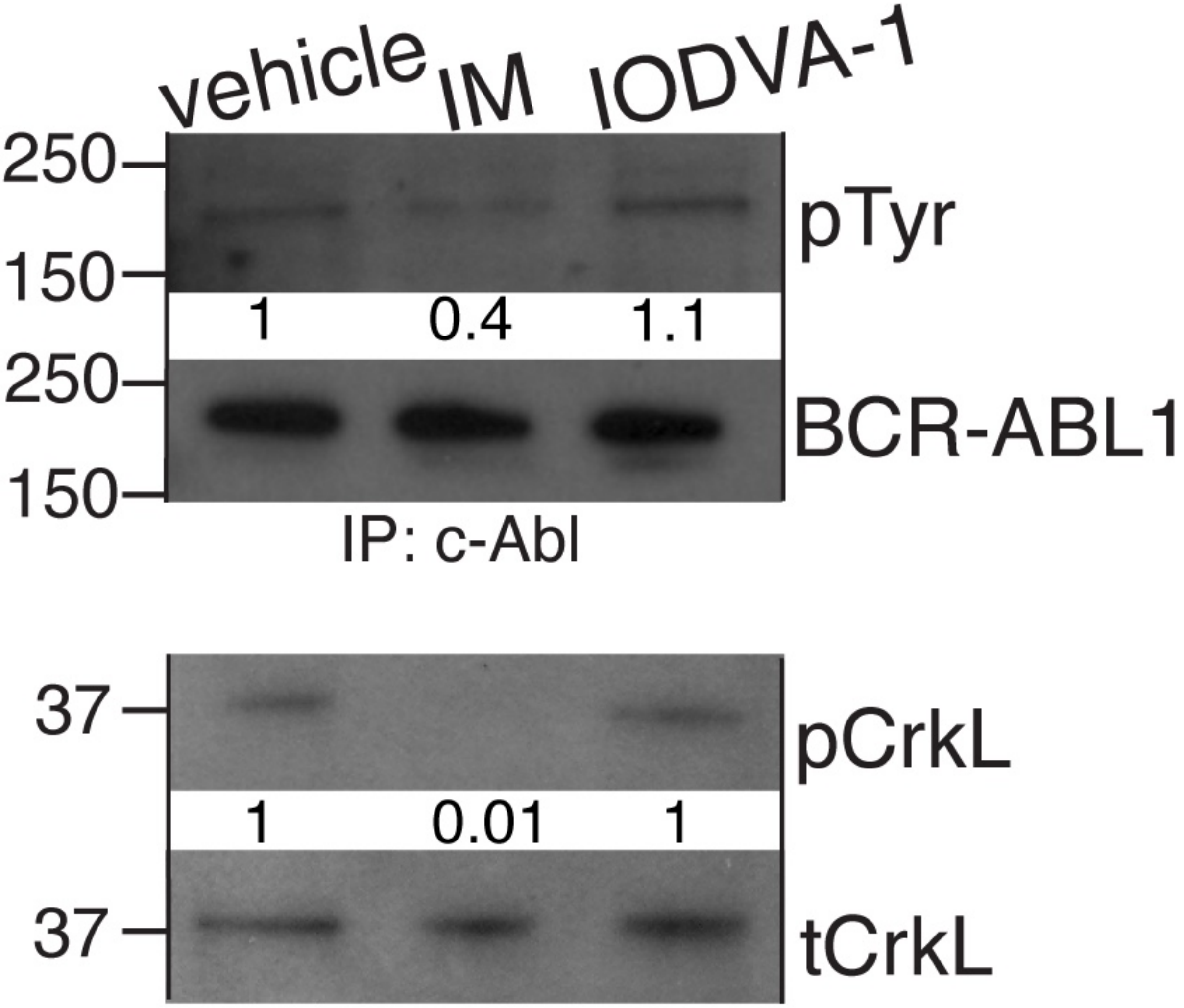
vehicle
IM
IODVA-1
250
150
pTyr
1
0.4
1.1
250
BCR-ABL1
150
IP: c-Abl
37
pCrkL
1
0.01
1
37
tCrkL

Figure S3

A Patient samples with Ph$^+$ rearrangements

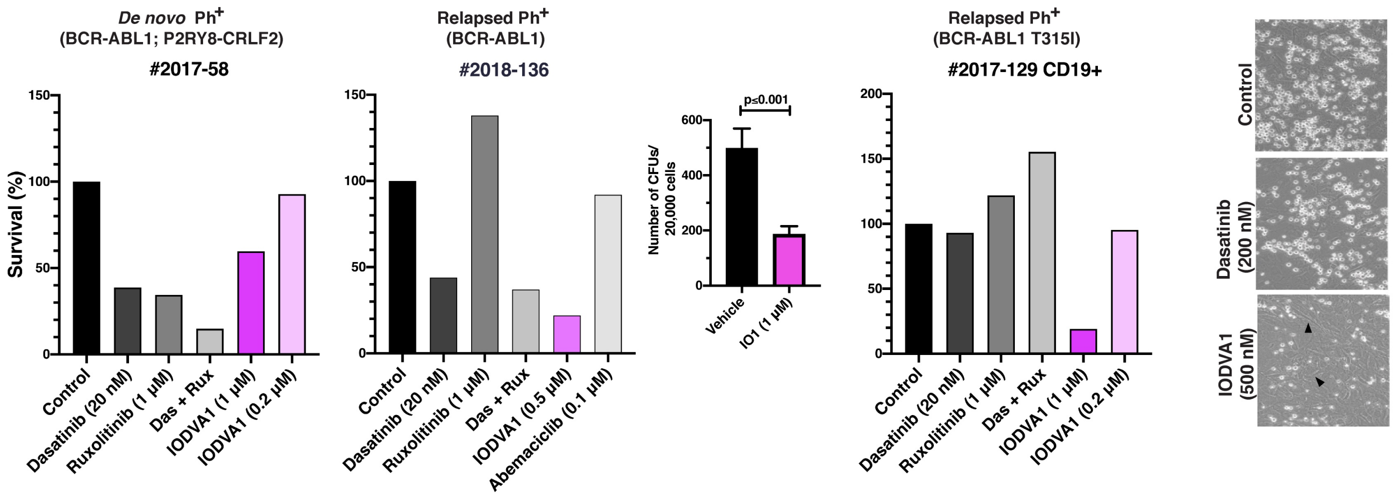

B Patient samples with *de novo* Ph-like rearrangements

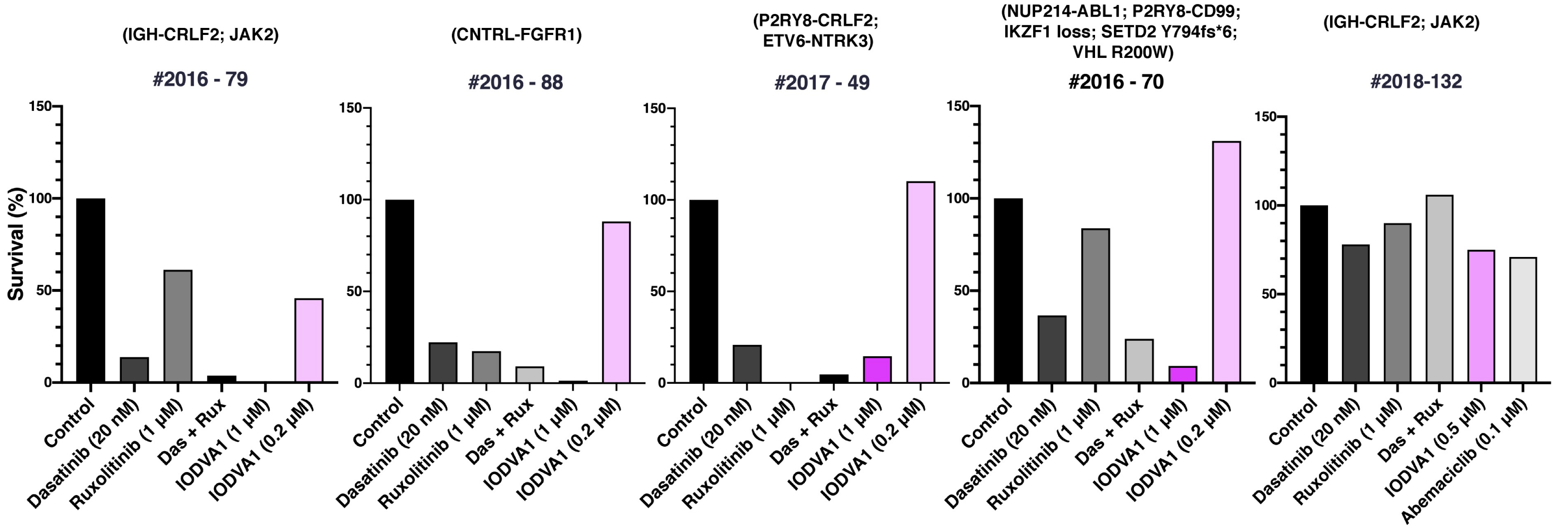

C Patient samples with MLL rearrangements

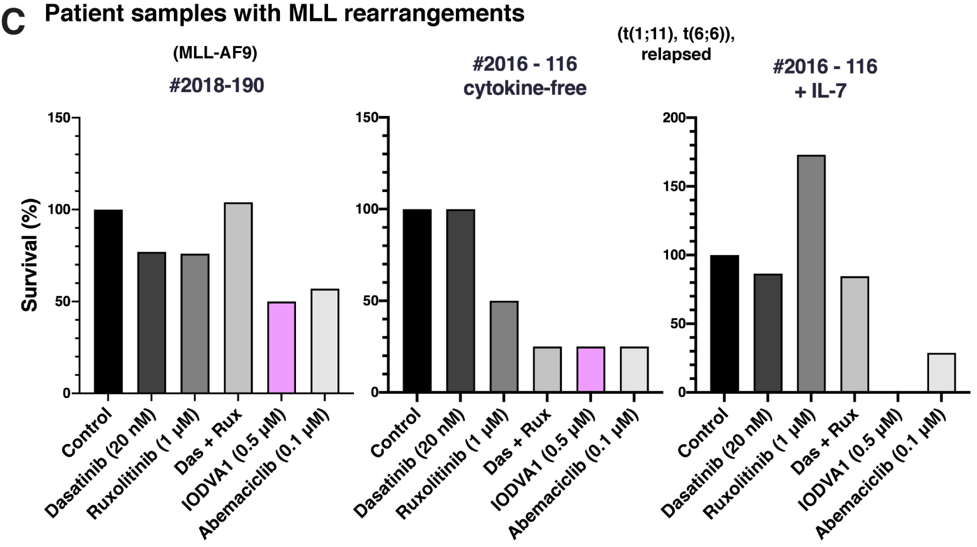

D

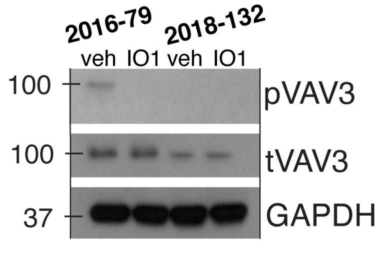

Figure S4

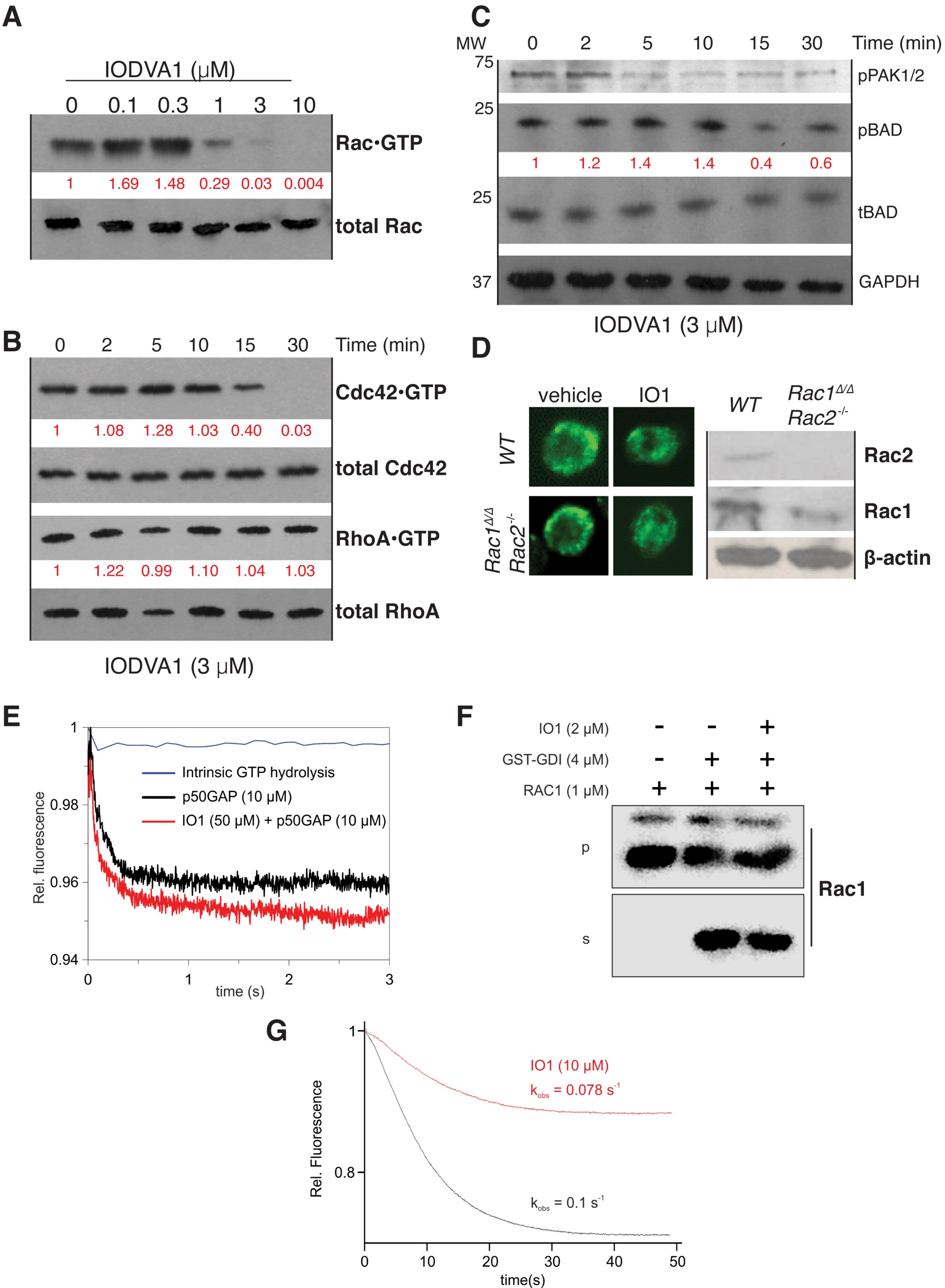
A
IODVA1 (μM)
0 0.1 0.3 1 3 10
Rac•GTP
1 1.69 1.48 0.29 0.03 0.004
total Rac
B
0 2 5 10 15 30 Time (min)
Cdc42•GTP
1 1.08 1.28 1.03 0.40 0.03
total Cdc42
RhoA•GTP
1 1.22 0.99 1.10 1.04 1.03
total RhoA
IODVA1 (3 μM)
C
MW 0 2 5 10 15 30 Time (min)
75
pPAK1/2
25
pBAD
1 1.2 1.4 1.4 0.4 0.6
25
tBAD
37
GAPDH
IODVA1 (3 μM)
D
vehicle IO1
WT
Rac1Δ/Δ Rac2-/-
WT
Rac1Δ/Δ Rac2-/-
Rac2
Rac1
β-actin
E
Rel. fluorescence
Intrinsic GTP hydrolysis
p50GAP (10 μM)
IO1 (50 μM) + p50GAP (10 μM)
1
0.98
0.96
0.94
0 1 2 3
time (s)
F
IO1 (2 μM) - - +
GST-GDI (4 μM) - + +
RAC1 (1 μM) + + +
p
s
Rac1
G
Rel. Fluorescence
1
0.8
IO1 (10 μM)
kobs = 0.078 s-1
kobs = 0.1 s-1
0 10 20 30 40 50
time(s)

Figure S5

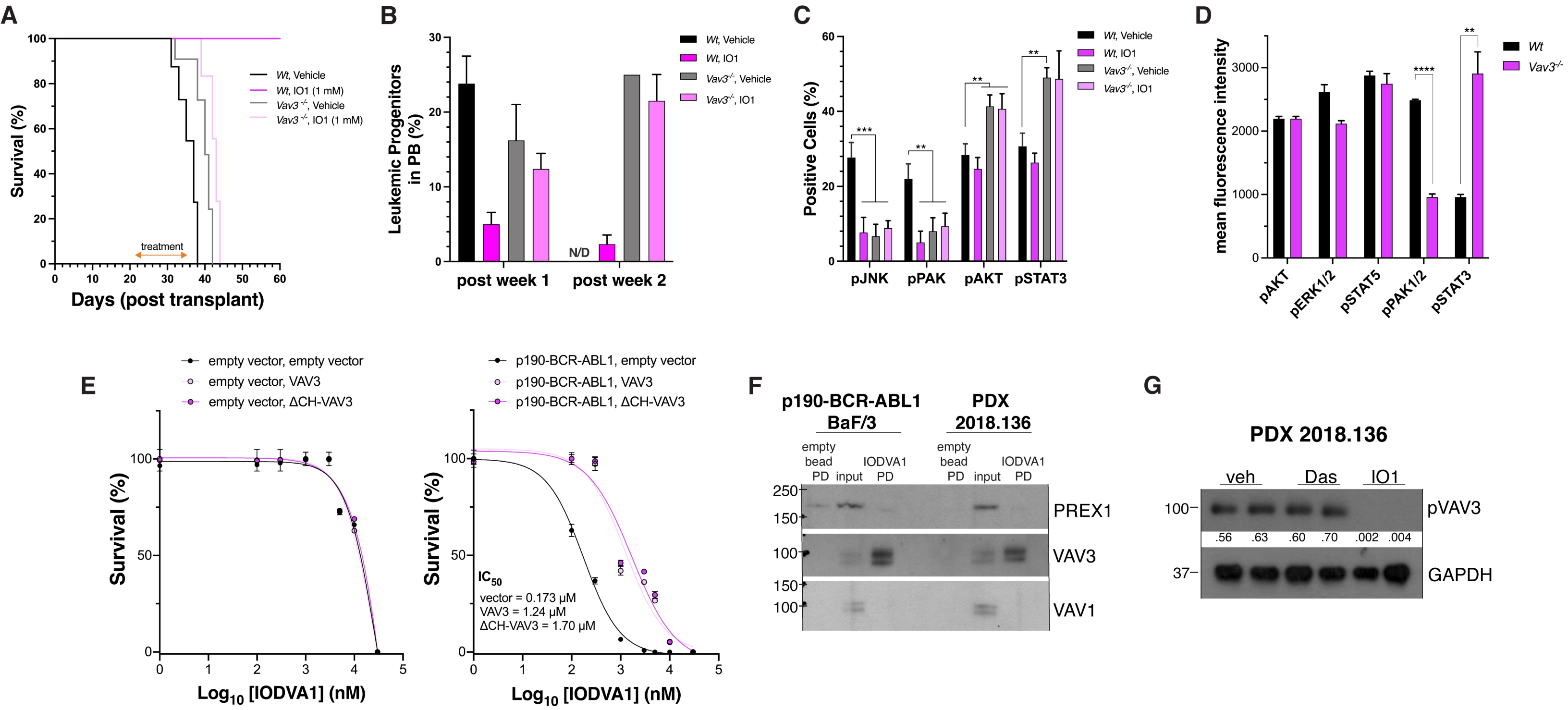

A
Survival (%)
Days (post transplant)
treatment
Wt, Vehicle
Wt, IO1 (1 mM)
Vav3 -/-, Vehicle
Vav3 -/-, IO1 (1 mM)
B
Leukemic Progenitors in PB (%)
post week 1
post week 2
N/D
Wt, Vehicle
Wt, IO1
Vav3-/-, Vehicle
Vav3-/-, IO1
C
Positive Cells (%)
pJNK
pPAK
pAKT
pSTAT3
D
mean fluorescence intensity
Wt
Vav3-/-
pAKT
pERK1/2
pSTAT5
pPAK1/2
pSTAT3
E
empty vector, empty vector
empty vector, VAV3
empty vector, ΔCH-VAV3
p190-BCR-ABL1, empty vector
p190-BCR-ABL1, VAV3
p190-BCR-ABL1, ΔCH-VAV3
Survival (%)
Log10 [IODVA1] (nM)
IC50
vector = 0.173 μM
VAV3 = 1.24 μM
ΔCH-VAV3 = 1.70 μM
F
p190-BCR-ABL1 BaF/3
PDX 2018.136
empty bead PD
input
IODVA1 PD
PREX1
VAV3
VAV1
G
PDX 2018.136
veh
Das
IO1
pVAV3
.56
.63
.60
.70
.002
.004
GAPDH

Table S1

PDX ALL models				
Patient #	Sample ID	Disease Stage	Mutations/Group	TKI History
ALL-009	2016-116 *	R/R	t(1;11), t(6;6)	
ALL-011	2016-79	De Novo	Ph-like (IGH-CRLF2; JAK2)	Ruxolitinib
ALL-012	2016-88	De Novo	Ph-like (CNTRL-FGFR1)	
ALL-014	2017-49	De Novo	Ph-like (P2RY8-CLRF2; ETV6-NTRK3)	Larotrectinib
ALL-015	2017-58 *	De Novo	Ph+ (BCR-ABL1); Ph-like (P2RY8-CRLF2)	Dasatinib
ALL-017	2017-129 *	R/R	Ph+ (BCR-ABL1, T315I)	Ponatinib
ALL-019	2018-132	De Novo	Ph-like (IGH-CRLF2; JAK2)	
ALL-004	2018-136	R/R	Ph+ (BCR-ABL1)	
ALL-032	2018-190	De novo	B-ALL (MLL/AF9)	
ALL-011	2016-70	De novo	Ph-like (NUP214/ABL1, IKZF-1, P2RY8/CD99, SETD2, VHL)	
